# Biophysical Network Modelling of the dLGN Circuit: Different Effects of Triadic and Axonal Inhibition on Visual Responses of Relay Cells

**DOI:** 10.1371/journal.pcbi.1004929

**Published:** 2016-05-20

**Authors:** Thomas Heiberg, Espen Hagen, Geir Halnes, Gaute T. Einevoll

**Affiliations:** 1 Department of Mathematical Sciences and Technology, Norwegian University of Life Sciences, Ås, Norway; 2 Institute of Neuroscience and Medicine (INM-6) and Institute for Advanced Simulation (IAS-6) and JARA BRAIN Institute I, Jülich Research Centre, Jülich, Germany; 3 Department of Physics, University of Oslo, Oslo, Norway; University College London, UNITED KINGDOM

## Abstract

Despite its prominent placement between the retina and primary visual cortex in the early visual pathway, the role of the dorsal lateral geniculate nucleus (dLGN) in molding and regulating the visual signals entering the brain is still poorly understood. A striking feature of the dLGN circuit is that relay cells (RCs) and interneurons (INs) form so-called triadic synapses, where an IN dendritic terminal can be simultaneously postsynaptic to a retinal ganglion cell (GC) input and presynaptic to an RC dendrite, allowing for so-called triadic inhibition. Taking advantage of a recently developed biophysically detailed multicompartmental model for an IN, we here investigate putative effects of these different inhibitory actions of INs, i.e., triadic inhibition and standard axonal inhibition, on the response properties of RCs. We compute and investigate so-called area-response curves, that is, trial-averaged visual spike responses vs. spot size, for circular flashing spots in a network of RCs and INs. The model parameters are grossly tuned to give results in qualitative accordance with previous in vivo data of responses to such stimuli for cat GCs and RCs. We particularly investigate how the model ingredients affect salient response properties such as the receptive-field center size of RCs and INs, maximal responses and center-surround antagonisms. For example, while triadic inhibition not involving firing of IN action potentials was found to provide only a non-linear gain control of the conversion of input spikes to output spikes by RCs, axonal inhibition was in contrast found to substantially affect the receptive-field center size: the larger the inhibition, the more the RC center size shrinks compared to the GC providing the feedforward excitation. Thus, a possible role of the different inhibitory actions from INs to RCs in the dLGN circuit is to provide separate mechanisms for overall gain control (direct triadic inhibition) and regulation of spatial resolution (axonal inhibition) of visual signals sent to cortex.

## Author Summary

While the basic receptive-field structure of cells in the dorsal lateral geniculate nucleus (dLGN), the station between retina and visual cortex in the early visual pathway, was mapped out half a century ago, the function of this nucleus in molding the visual signals is still poorly understood. One reason is that the dLGN contains enigmatic inhibitory interneurons which can act with different inhibitory action on the excitatory relay cells. In addition to standard axonal inhibition, relay cells and interneurons form so-called triadic synapses, where an interneuron dendritic terminal can be simultaneously postsynaptic to a retinal input and presynaptic to a relay-cell dendrite, opening up for so-called triadic inhibition. Taking advantage of a recently developed biophysically detailed multicompartmental model for an interneuron, we here use a network model to investigate putative effects of these inhibitory actions on the response properties of relay cells stimulated by circular flashing spots. Our results suggest a possible role of the different inhibitory actions in providing separate mechanisms for overall gain control (triadic inhibition) and regulation of spatial resolution (axonal inhibition) of visual signals sent to cortex.

## Introduction

The dorsal lateral geniculate nucleus (dLGN) acts as a gateway for visual signals that reach cortex. The principal cells, the relay cells (RCs), constitute about 75–80% of the cells in the nucleus, while the remaining 20–25% are intrageniculate interneurons (INs) [1]. The RCs receive synaptic inputs from a variety of sources: direct *feedforward excitation* from retinal ganglion (GC) cells [2–8], indirect *feedforward inhibition* via the INs, which in turn are excited by GC cells [7, 9], *feedback inhibition* from the thalamic reticular nucleus (TRN) [1] and *feedback excitation* from primary visual cortex [10, 11]. Both the IN and TRN cells further receive excitatory feedback from cortex opening up for *feedback inhibition* of RCs involving the entire thalamocortical loop [1]. Despite its prominent position in the early visual pathway, and the relative abundance of anatomical and physiological data recorded from the nucleus, the functional role of the dLGN circuit is still poorly understood. Mathematical modeling of the properties of the network will clearly have to be a key component in elucidating its function.

A striking feature of the dLGN circuit is that INs and RCs are known to form so-called triadic synapses [12–16]. Such triadic synapses are typically formed at sites that are proximal on the RC dendrites and distal on the IN dendrites. At these sites, a single retinal terminal contacts postsynaptic terminals on both an IN dendrite and an RC dendrite. The IN terminal is, at the same time, postsynaptic to the GC input and presynaptic to the RC [14]. In the triads, GABA-release from the IN may be triggered directly by local GC input, providing a localized source of inhibition of RCs, which may be functionally decoupled from the IN soma [12, 13, 15, 16]. In addition to the complex triadic action, the INs also provide standard, axonal inhibition of RCs [14].

Until now, there has to our knowledge been no dLGN network study investigating the functional role of these triadic circuit elements. A key reason is that while several biophysically detailed neuron models for RCs have been developed [17–23], models of INs have been more scarce. However, recently our group developed the first comprehensive multicompartmental IN models including active dendritic conductances placed on anatomically reconstructed dendritic morphologies [24], opening up for investigations of the functional role of the different putative inhibitory action by INs on RCs in the dLGN network.

Various types of visual stimuli have been used to probe the response properties of the dLGN circuit: light or dark bars, gratings, and spots of various sizes [25]. Based on experiments with flashing circular spots [26], Einevoll and Heggelund [27] developed a mechanistic firing-rate model to account for the changes in the spatial response properties of RC cells in cat compared to its GC input. In qualitative accordance with known anatomy and physiology for cat X cells, the RC neurons in the model received excitatory input from single GC neurons and indirect feedforward inhibition from INs, which in turn received input from of a handful of GC neurons. While this model successfully accounted for the observed area-summation curves in RC cells, i.e., the experimentally observed response vs. spot-diameter curves, it could not distinguish between the various possibilities of inhibitory action from INs to RCs, i.e., whether the inhibition was predominantly triadic or axonal.

To investigate the putatively different roles of triadic and axonal inhibitory action from INs in the dLGN circuit, we here develop and investigate a biophysically detailed, spiking neuron network model designed to be analogous to the firing-rate network model in [27]. A key component of the network is an adapted version of the recent multicompartment IN model [24] allowing for explicit studies of how the various modes of inhibition affect the shape of measured spot-response curves for dLGN cells [26].

In the next section we introduce the circuit model and describe the models of the GC input, the IN and the RC, as well as their synaptic connections. In Results we first investigate and describe the behavior of the IN model, then probe the functional behavior of the triadic circuit. Next, we illustrate how the various modes of inhibition affect the area-summation curves and finally explore differences between the transient (onset) and sustained (steady-state) responses to spot stimulation. Our findings are then discussed in the final Discussion.

## Materials and Methods

### dLGN circuit model

**Fig 1:**
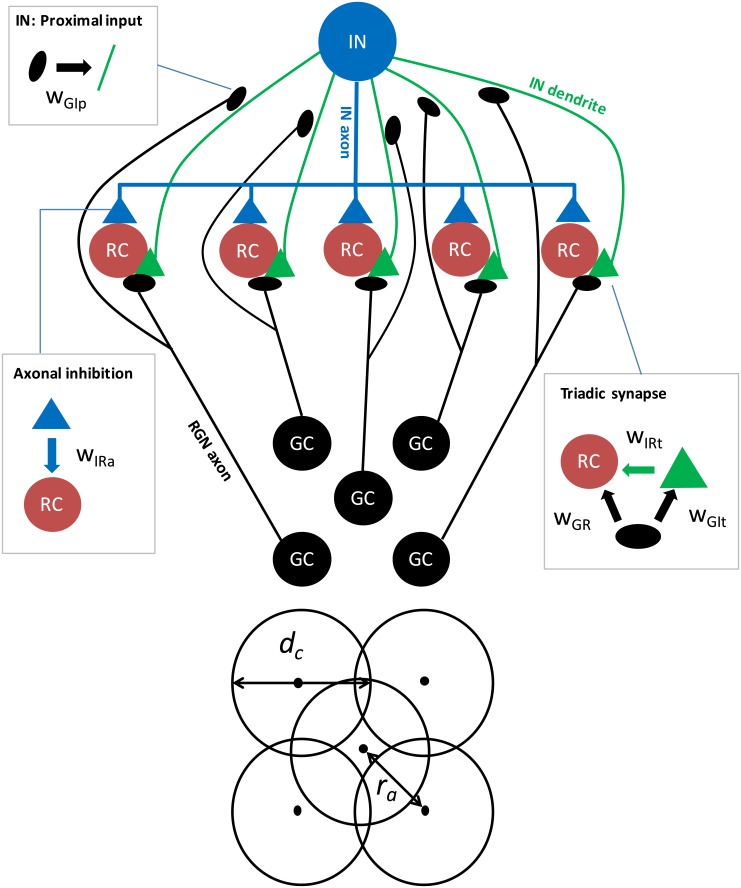
Schematic of the dLGN circuit model. (Top) Five relay cells (RCs) receive input from one retinal ganglion (GC) cell each. All inputs to RCs arrive in triadic synapses, involving the one and same IN. In addition, the IN receives proximal input from all five GCs. The boxes highlight the synaptic connections in the networks and the associated connection weights *w*. Note that in the present model application, only responses for the central RC cell is considered so that the only effect of the four peripheral GCs comes from the proximal inputs to the IN. (Bottom) The GCs are organized with four peripheral GCs all located at distance *r*_a_ from the center GC.

Input to the dLGN circuit was provided by a layer of five retinal ganglion neurons (GCs), spatially organized with one center cell and four peripheral cells equidistant from the center cell (Fig 1). Each GC axon was assumed to synapse at two different locations, i.e., (i) in a triadic synapse where the interneuron (IN) and one of the relay cells (RCs) both receive excitatory input, and (ii) in a ‘conventional’ synapse on the proximal IN dendrite. The IN formed two inhibitory synapses on each of the five RCs, (i) a dendrodendritic synapse (part of the triad) and (ii) an axodendritic synapse.

In the present application of the model we only computed the response of the central RC. In addition to the local triadic inhibitory action due to synaptic inputs from the central GC (called *direct triadic inhibition* below), this cell received extra ‘back-propagating’ triadic inhibition (called *soma-driven triadic inhibition* below) and axonal inhibition following firing of action potentials in the IN. Thus the RCs were decoupled in the sense that firing of action potentials in one RC did not affect the firing of the other RCs. Therefore, the only effect of the four peripheral (non-central) GCs came from their proximal inputs to the IN. For simplicity we here assumed that these four synaptic weights are the same, an approximation which is unlikely to bear out in real biological situations. However, the use of circular flashing spot stimuli concentric with the receptive field of the central RC, implies that the response of the central RC will largely be determined by the *sum* of these four weights, not their individual variation [27].

The spike trains of GCs were modeled descriptively as non-stationary Poisson processes. The visual input driving the GCs were circular light spots centered on the middle GC. The outputs were spike trains with mean rate and temporal profile fitted to experimental data.

The components that make up our circuit were modeled at different levels of detail. To allow for local processing in the dendrites and because the IN is known to be electrotonically extensive [28], a multicompartment model was needed. We selected an existing model [24] and simplified its morphology. Some of the parameters were adjusted to otherwise preserve the model’s properties.

The RC spikes constitute the main output from our network model. A single-compartment RC model was decided to be sufficient as these neurons are thought to be electrotonically compact [28]. With slight modifications discussed below, a previously published model was used [29].

The IN and RC models were both based on standard cable theory (see e.g., [30]), and the complete dLGN circuit model was implemented in the NEURON simulation environment [31–33]. Both neuron models were based on previously published models and are available from ModelDB [34]: IN model from [24] (ModelDB accession number 140249) and RC model from [29] (ModelDB accession number 3343).

In the following section, the individual components of the circuit and their parameterizations are presented in detail.

### Input from retinal ganglion (GC) cells

As in the firing-rate based circuit model of [27], a descriptive filter model was used to generate the input from the GC cells to our model dLGN circuit. Specifically, the input spike trains from the five GC cells were generated by non-stationary Poisson processes with rates determined by a response function *R*_g_(*t*, *d*) describing the firing rate for a circular spot of radius *d* as a function of time. This response function was in turn modeled as a product over a spatial part *G*_g_(*d*) and a temporal part *F*_g_(*t*) [35], i.e., *R*_g_(*t*, *d*) = *G*_g_(*d*)*F*_g_(*t*).

#### Spatial part of GC input response function

Following [27] we modeled the shape of the spatial receptive-field (point-spread) functions *g*_g_(*r*) by means of the difference-of-Gaussians (DOG) model [36],

$$\begin{array}{c} g_{\text{g}}\left(r\right)=\frac{1}{\pi a_{1}^{2}}e^{-r^{2}/a_{1}^{2}}-\frac{\omega }{\pi a_{2}^{2}}e^{-r^{2}/a_{2}^{2}}\;\;, \end{array}$$ (1)

where the first and second terms term correspond to the center and surround terms, respectively. Further, *ω* represents the relative strengths of these terms, and *a*_1_ and *a*_2_ are the corresponding width parameters.

We further assume that the total neuronal response is given as a sum of the inputs caused by the spot with luminance *L*_spot_ and the infinite background surrounding the spot with luminance *L*_bkg_. For the single GC cells with receptive-field center concentric with the spot stimulus (see Fig 1), the response function is then found to be [27]:

$$\begin{array}{ccc} \;\;\;\;\;\;\;\;\;\;\;\;\;G_{\text{g}}\left(d;0\right) & = & S[\;l_{\text{bkg}}\left(1-\omega \right)\\ \;\;\;\;\;\;\;\;\;\;\;\;\; & + & \left(l_{\text{spot}}-l_{\text{bkg}}\right)\left(1-e^{-d^{2}/4a_{1}^{2}}-\omega \left(1-e^{-d^{2}/4a_{2}^{2}}\right)\right)\;]\; \end{array}$$ (2)

where the halfwave rectification function *S*[*x*] = *x*Θ(*x*) has been introduced to enforce non-negative firing rates. Here Θ(*x*) is the Heaviside step function, and an activity function *l*(*L*) converting luminance to firing rates has been introduced, i.e., *l*_bkg_ ≡ *l*(*L*_bkg_), *l*_spot_ ≡ *l*(*L*_spot_). The ‘0’ in the notation *G*_g_(*d*;0) signifies that the spot and receptive fields are concentric, i.e., a distance zero between their centers.

Four of the GC neurons driving the dLGN circuit have receptive fields that are not concentric with the spot, however. Rather, their receptive field centers are displaced a distance *r*_a_ from the spot center (Fig 1). In this situation the spot-response function is instead given by [27]

$$\begin{array}{ccc} G_{\text{g}}\left(d;r_{\text{g}}\right) & = & \;\;S[\;l_{\text{bkg}}\left(1-\omega \right)+\left(l_{\text{spot}}-l_{\text{bkg}}\right)\times \\ & & (\;e^{-r_{\text{a}}^{2}/a_{1}^{2}}\sum_{m=0}^{\infty }\frac{1}{m!}{\left(\frac{r_{\text{a}}}{a_{1}}\right)}^{2m}\gamma \left(m+1,d^{2}/4a_{1}^{2}\right)\\ & & -\omega \;e^{-r_{\text{a}}^{2}/a_{2}^{2}}\sum_{m=0}^{\infty }\frac{1}{m!}{\left(\frac{r_{\text{a}}}{a_{2}}\right)}^{2m}\gamma \left(m+1,d^{2}/4a_{2}^{2}\right)\;)\;]\; \end{array}$$ (3)

where *γ*(*n*, *x*) is the so-called incomplete gamma function given by

$$\begin{array}{c} \gamma \left(n,x\right)=\frac{1}{(n-1)!}\int_{0}^{x}u^{m-1}e^{-u}\;du \end{array}$$ (4)

when *n* is an integer larger than zero. Note that for *r*_a_ = 0, Eq (3) simplifies to Eq (2). Note also that since we only consider visual stimuli with circular symmetry, i.e., circular spots, the model response does not depend on the perfect square arrangement of the non-concentric GC inputs as depicted in Fig 1B. The magnitude of a particular peripheral GC input only depends on the distance *r*_a_ from the central GC cell.

The spatial characteristics of the GC inputs to the circuit can thus be parameterized by the GC parameters *ω*, *l*_bkg_, *l*_spot_, *a*_1_, and *a*_2_, as well as the distance between central and peripheral GC centers *r*_a_. Here we assumed the five GC neurons providing the inputs to the dLGN circuit to have the same response properties, i.e., the same values of *ω*, *l*_bkg_, *l*_spot_, *a*_1_, and *a*_2_. The parameters used here were found in [27] from fitting the GC response function in Eq (2) to experimental data in [27] (see cell no. 2 depicted in Fig 5 therein). This parameterization was selected because it is close to the mean of the results reported there and also the parameterization used in examples throughout that paper. The parameters are listed in Table 1.

**Table 1:** Model parameters for input from retinal ganglion cells (GCs).

Parameter	Description	unit	value
*ω*	relative strength between surround and center		0.85
*l*_bkg_(1 − *ω*)	activity function (background)	*s*^−1^	36.8
*l*_spot_(1 − *ω*)	activity function (spot)	*s*^−1^	56.5
*a*_1_	center width	deg	0.62
*a*_2_	surround width	deg	1.26
*r*_a_	peripheral GC receptive-field center displacement	deg	0.99
*τ*_1_	time constant of first exponential	ms	10.0
*τ*_2_	time constant of second exponential	ms	22.0
*α*	global scaling		12.0
*β*	relative scaling of second exponential		11.26

#### Temporal part of GC input response function

The temporal profile of the GC spike trains was modeled as a difference of two exponential functions,

$$\begin{array}{c} F_{\text{g}}\left(t\right)=\Theta \left(t-t_{\text{s}}\right)\;\alpha \left(1-e^{-\left(t-t_{\text{s}}\right)/\tau_{1}}-\beta \left(1-e^{-\left(t-t_{\text{s}}\right)/\tau_{2}}\right)\right) \end{array}$$ (5)

to incorporate the overshoot seen in experiments (e.g. [26], see Fig 3 and 4 therein) following stimulus onset (or more precisely onset of stimulus-evoked response in the GCs in our model) at time *t*_*s*_. The parameters (see Table 1) were chosen to approximate the magnitude and width of the peak in the experiments of [26] (see Fig 4B therein), with a maximum of about 2.5 times the sustained rate, i.e., the firing rate long after stimulus onset, while retaining the mean rate for the stimulus period given by the spatial response function.

### Interneuron model

#### Model and calibration

An adapted and simplified version of the multicompartmental IN model by [24] was used. In particular, we employed a simplified dendritic morphology consisting of a cylindrical soma (with radius 8.72 *μ*m and length 15.3 *μ*m) with five identical linear ‘stick’-like dendrites protruding out from it. These dendrites had linearly tapered diameters going from 4 *μ*m adjacent to the soma to 0.3 *μ*m at a distance of 100 *μ*m, and from there on a constant diameter up to a total length of 500 *μ*m.

We employed a set of passive membrane properties and active channel conductances with corresponding kinetics from [24] (Parameter set 1). The seven active ion-channels included the traditional Hodgkin-Huxley sodium and delayed-rectifier potassium channels (with conductances *g*_Na_ and *g*_Kdr_, respectively), a hyperpolarization-activated cation channel (*g*_h_), a low-threshold, T-type calcium channel (*g*_CaT_), a high-threshold, L-type calcium channel (*g*_CaL_), a medium-duration, calcium-dependent afterhyperpolarization channel (*g*_AHP_), and a long-lasting calcium-activated non-specific cation channel (*g*_CAN_). The intracellular Ca^2+^-concentration was modeled as a leaky integrator [24].

To adjust for the simpler morphology compared to the morphologies used in [24], and account for recent experimental findings, some parameter values were modified: (i) The reversal potential of the passive leak current (*E*_pas_) was modified to adjust the resting membrane potential, which was kept at -63 mV. (ii) The dendritic conductances *g*_Na_ and *g*_Kdr_ were set so that a somatically generated action potential (AP) reliably invaded distal dendrites (backpropagating APs), while synaptically evoked AP propagation from distal dendrites to the soma reliably failed. This was done to accommodate recent experimental findings [37]. (iii) In [24], *g*_CaT_ was set to increase linearly with distance from soma. However, with high values for *g*_CaT_ in distal dendrites, synaptic activation was likely to induce Ca^2+^ spikes and bursts of APs that originated locally in distal dendrites [38]. Such effects were not observed in experimental studies of dendritic signalling [37]. We here therefore assumed that *g*_CaT_ and *g*_CAN_ were uniformly distributed over the dendritic membrane, as this significantly reduced locally induced AP-firing in the dendrites. A uniform distribution of *g*_CaT_ also agreed better with another experimental study, which showed that somatically elicited Ca^2+^-spikes evoked Ca^2+^ transients that were of the same magnitude across the entire dendritic tree [37]. (iv) In [24], *g*_CAN_ was assumed to have the same distribution as *g*_CaT_. Here, we kept this assumption, and used a uniform distribution also for *g*_CAN_.

**Fig 2:**
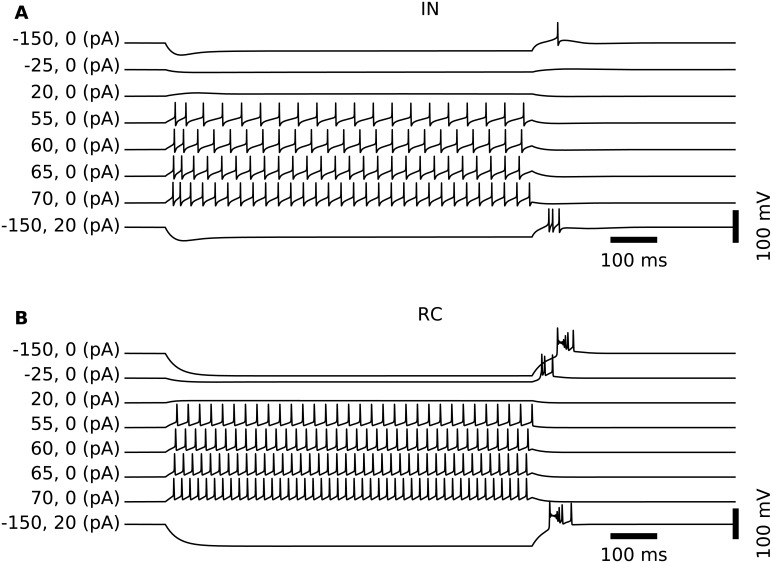
Spiking patterns of model neurons following somatic current injection. (A) Somatic membrane potentials of the IN model following injection of depolarizing and hyperpolarizing (positive and negative values, respectively, of first of two numbers in parenthesis) step currents lasting 900 ms. Results illustrate the overall tonic-firing response to depolarizing input currents. For the case with a strong hyperpolarizing current (−150 pA), a rebound spike is observed at offset (top trace). In the case where the offset of the strong hyperpolarizing step current (−150 pA) is combined with a constant but weak depolarizing current (+20 pA), a rebound burst is observed instead (bottom trace). (B) Similar to the IN cell, the RC cell generates spikes in a tonic pattern when the soma receives depolarizing currents. However, compared to the IN, the RC cells respond with more spikes for similar-amplitude depolarizing soma currents (and also more rebound spikes after offset of hyperpolarizing currents).

Model parameters for passive and active membrane properties for the somatic compartment and dendritic sections are summarized in Table 2. With these parameters, the simplified IN model preserved the qualitative response properties of the original model to somatic current injection [24]. Further, the resting membrane potential of this IN model is –63 mV, and for this membrane-potential value the model responded to depolarizing current injections into the soma with tonic AP-firing, with a slightly higher firing rate immediately after current onset, see Fig 2. This resembles the tonic firing mode described for dLGN cells [39].

**Table 2:** Interneuron (IN) parameters.

Parameter	Description	unit	soma	dendrites
*r*_ax_	axial resistivity	Ω⋅cm	113	113
*c*_m_	membrane capacitance	*μ*F/cm^2^	1.1	1.1
*r*_m_	membrane resistivity	Ω⋅cm^2^	22000	22000
*E*_pas_	passive leak reversal potential	mV	-67.5	-67.5
*g*_Na_	max Na^+^ conductance	S/cm^2^	0.1	0.0074
*E*_Na_	Na^+^ reversal potential	mV	50	50
SH_Na_	Na activation threshold	mV	-52.6	-52.6
*g*_K, dr_	max K_dr_ conductance	S/cm^2^	0.37	0.037
SH_K, dr_	K_dr_ activation threshold	mV	-51.2	-51.2
*E*_K_	K^+^ reversal potential	mV	-90	-90
*g*_CaT_	max CaT conductance (permeability)	cm/s	1.8⋅10^−4^	1.8⋅10^−4^
*g*_CaL_	max CaL conductance (permeability)	cm/s	9.0⋅10^−4^	2.25⋅10^−4^
[Ca]	basal Ca^2+^ concentration	nM	50	50
*τ*_Ca_	Ca^2+^ decay time constant	ms	50	50
*g*_AHP_	max *I*_AHP_ conductance	S/cm^2^	6.4⋅10^−5^	6.4⋅10^−6^
*g*_CAN_	max *I*_CAN_ conductance	S/cm^2^	6.8⋅10^−7^	6.8⋅10^−7^
*g*_h_	max *I*_h_ conductance	S/cm^2^	1.1⋅10^−4^	1.1⋅10^−4^

#### Input

Each GC was assumed to synapse onto the IN in two spatially separated locations, contacting the IN dendrites (i) at the proximal IN synapse (50 *μ*m from the soma; weight denoted *w*_GIp_), and (ii) in the triadic synapse located at the distal IN dendrite (450 *μ*m from the soma; weight denoted *w*_GIt_). Each GC projected to one of the five dendritic sections on the IN unit.

Conductance-based synapses were assumed, i.e.,

$$\begin{array}{c} I_{\text{syn}}\left(t\right)=wf_{\text{syn}}\left(t\right)\left(V-E_{\text{syn}}\right)\;, \end{array}$$ (6)

where the weight *w* corresponds to the maximal conductance, and the temporal envelope *f*_syn_(*t*) of the synaptic conductance is given as the difference between two exponentially decaying functions specified by rise (*τ*_rise_) and decay (*τ*_decay_) times and normalized so that the maximum value of *f*_syn_(*t*) is unity, cf. Eqs. 6.4–6.6 in [40].

The properties of the proximal synapse were adapted to give responses in accordance with experimental data where EPSPs have been found to be dependent on AMPA and NMDA activation, but not on mGluR activation [41]. The joint AMPA and NMDA response was modeled as a sum of two exponentials [40, ch.6]. We used an AMPA reversal potential of 10 mV [15], and adapted the time constants of synaptic rise and decay, as well as maximum conductance to *in vitro* EPSC-traces in [41]. With these values, the time course of the somatic EPSCs in INs resembled those observed experimentally. We adjusted the synaptic weights so that the IN model required simultaneous activation of both the proximal and distal synapses on four dendrites in order to produce an action potential. This agrees with experiments, where typically 3–4 simultaneous synapse activations were required to evoke action potentials in INs [41]. The synaptic parameters are summarized in Table 3.

**Table 3:** Synaptic parameters, cf. Eq 6. The listed parameters for the weights *w*_GR_, *w*_GIp_, *w*_IRt_, and *w*_IRa_ are only the default values, other values are also considered, cf. Table 5. The other parameters are kept fixed in the study.

Presyn.	Postsyn.	Weight label	*w* (nS)	*E*_syn_ (mV)	*τ*_rise_ (ms)	*τ*_decay_ (ms)
GC	IN triad	*w*_GIt_	2	10	0.3	2.0
GC	RC	*w*_GR_	11.6	10	0.2	1.2
GC	IN proximal	*w*_GIp_	0.6	10	1.6	3.6
IN triad	RC	*w*_IRt_	4	-80	0.7	4.2
IN axon	RC	*w*_IRa_	4	-80	0.7	4.2

The response properties of the distal synapse (IN-side of triadic synapse) was initially modeled after [41] like the proximal synapses. However, in the triad the parameters were adjusted so that the triad supported so-called ‘locked’ (i.e., ‘time-locked’) inhibition of RC cells following the excitatory GC input input spike by ∼1 ms [15], see below. As triadic synapses are located in the distal part of IN dendrites, triadic synaptic activation were found not have any strong impact on the membrane potential in the soma of the IN (postsynaptic potential amplitudes ∼1 mV)

#### Output

Axonal GABA release from INs was assumed to occur whenever the soma elicited an AP, detected by somatic voltage crossings at –10 mV, with a 1 ms conduction delay. All five relay cells were contacted by the axon, and received the same axonal inhibition (although only the inhibition of the central RC was of relevance in the present model application focusing solely on the response of the central RC).

It has been suggested that GABA release from IN dendrites in the triad is mediated by a depolarization of the presynaptic terminal [42]. We assumed that GABA release from dendritic sites was triggered whenever the local voltage exceeded a threshold of –10 mV. With this threshold, the model reproduced two independent experimental observations. Firstly, synaptic GC input to triadic terminals in most cases (yet dependent on the history the IN activity) resulted in local GABA release from IN terminals. RCs typically responded to triadic GC input by an EPSP (synaptic excitation) followed by an IPSP about 1 ms after, as has been observed experimentally and coined ‘locked’ inhibition [15]. We refer to this input-induced inhibition as *direct triadic* inhibition. Secondly, local GABA release could also in some instances be evoked by back-propagating action potentials or Ca^2+^ spikes with somatic origin [41]. We refer to this as *soma-driven triadic* inhibition.

### Relay-cell model

#### Model and parameter calibration

In contrast to INs, relay cells (RCs) appear to be electrotonically compact [28], and we thus use a single-compartment model. The membrane mechanisms were taken from an existing model [29], and included the standard (Hodgkin-Huxley type) sodium and potassium channels for generating action-potentials, as well as T-type Ca^2+^-channels. The conductances *g*_Na_, *g*_K_ and *g*_CaT_ were tuned so as to obtain qualitatively typical responses to somatic current injections for cells resting at a relatively depolarized membrane potential (–60 mV) [39] set by adjusting the reversal potential of the passive (leak) current (*E*_pas_). In this relatively depolarized state, the RC model responded to somatic current injections by tonic firing of spikes as shown in Fig 2. The final parameter set is summarized in Table 4.

**Table 4:** Model parameters for relay cell (RC).

Parameter	Description	unit	value
*L*	soma length	*μ*m	35
*d*	soma diameter	*μ*m	47
*c*_m_	membrane capacitance	*μ*F/cm^2^	1.0
*r*_m_	membrane resistivity	Ω⋅cm^2^	26000
*E*_pas_	passive leak reversal potential	mV	-63
*g*_Na_	max. Na conductance	S/cm^2^	0.015
*E*_Na_	Na reversal potential	mV	50
SH_Na_	Na activation threshold	mV	-50
*g*_K_	max. K conductance	S/cm^2^	0.0025
*E*_K_	K reversal potential	mV	-100
[Ca]	*t*_∞_ Ca^2+^-concentration	nM	240
*g*_CaT_	max. CaT conductance	S/cm^2^	0.001
SH_CaT_	Shift for ext. [Ca] = 2mM	mV	2
*E*_Ca_	Ca^2+^ reversal potential	mV	120

#### Input and output

As illustrated in Fig 1, the RC received (i) excitatory input from GCs in triadic synapses, (ii) inhibitory input from INs via dendritic GABA release in triadic synapses, and (iii) inhibitory input from INs via axonal GABA release.

The postsynaptic model response of RCs to glutamatergic input from the GCs was adapted to experimental data, i.e., monosynaptic excitation was assumed mediated by AMPA receptors with a reversal potential of 10 mV [15]. We constrained synaptic parameters (time constants, maximum conductance) to reproduce experimentally obtained EPSCs (Fig 4 in [15]).

The postsynaptic model response of RCs to dendritic GABA release from INs in the triadic synapses was adapted to experimental data on direct triadic (‘locked’) inhibition [15]. The synaptic response was assumed mediated by GABA_A_ receptors with a reversal potential of –80 mV. We constrained synaptic parameters (time constants, maximum conductance) to reproduce experimentally obtained IPSCs (Fig 4 in [15]).

In the experimental study of [15] the shapes of IPSCs produced by dendritic and axonal GABA-release were observed to be similar. We therefore modeled the synaptic response of RCs to axonal inhibition from INs to have the same functional shapes as for the triadic inhibition. Parameter values for all synapse models are summarized in Table 3.

### Stimulus protocol

Neuron and synapse parameters were initially set up according to the calibrated (default) parameters listed in Tables 1–4. As in [27] we modeled the response to circular spots concentric with the receptive field of the central GC input (cf. Fig 1). The only stimulus parameter varied was thus the spot diameter *d*, with the spot sizes ranging from much smaller than, to much larger than the receptive-field center. In the simulations each trial consisted of a 500 ms period of full-field background luminance followed by a 500 ms stimulus period with the circular spot added on top.

In accordance with [27], mean firing rates from GC, IN, and RC cells over the entire or selected parts of the stimulus period were computed. (These firing-rates were found from time-averaging post-stimulus time histograms (PSTHs) and correspond to what is more precisely referred to as ‘spike-count’ firing rates [43], but in the present paper we will for simplicity generally refer to them as firing rates.) However, all spike trains were also stored for further analysis. In addition, membrane potentials from relevant neural compartments (i.e., RC and IN soma compartments as well as IN triad compartments) were recorded for a subset of the trials.

**Fig 3:**
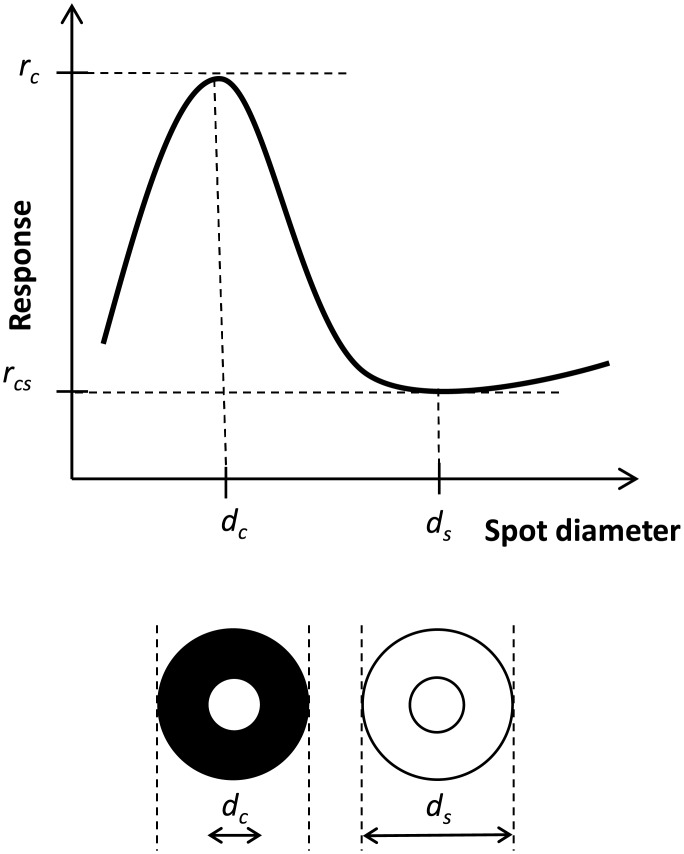
Illustration of area-response curves and metrics used to quantify key properties. Center diameter *d*_c_, surround diameter *d*_s_, peak response rate *r*_c_, center-surround minimum rate *r*_*cs*_. Illustration adapted from Fig 1 in [26].

For each spot diameter several simulations (‘trials’) were run, and the spike-count firing rate for each trial computed. So called area-summation response curves of the type considered in [26] and [27], i.e., spike-count firing rates averaged over numerous trials as functions of spot diameter, were produced (cf. Fig 3). Unless otherwise noted, ten trials were used in the computation of the trial-average firing rate for each parameter set and spot size, and the response vs. spot-diameter curves were filtered with a seven-point rectangular window to produce smoother area-summation curves. Such area-summation curves were calculated for a large set of parameter values (cf. Table 5) to investigate the link between model parameters and response curves.

**Table 5:** Parameter space explored for spot size and synaptic weights (maximal synaptic conductances, cf. Eq 6) in simulations. † denotes default values.

Parameter	Description	Unit	Values
*d*	Stimulus (spot) diameter	deg	0.05, 0.1, …, 10.0
*w*_GR_	GC → RC	nS	†11.6/13.6/15.6/17.6
*w*_GIp_	GC → IN proximal	nS	0.3/†0.6/1.2/1.8
*w*_IRt_	IN triad → RC	nS	0/†4
*w*_IRa_	IN axon → RC	nS	0/2/†4/6/8

### Analysis of simulation results

In the present application of the model we only considered the response of the IN and the central RC.

The receptive-field center diameter *d*_c_ was determined numerically by identifying the spot diameter that produced the maximum response *r*_c_, see Fig 3. Here we were interested both in maximal responses for the RC ($r_{c}^{R}$) and IN ($r_{c}^{I}$). Similarly, the surround diameter *d*_s_ was given by the spot size diameter producing the minimum response *r*_cs_, and at the same time fulfilling *d*_s_ > *d*_c_.

From these four quantities we calculated several response measures: The ratio $d_{\text{c}}^{\text{R}}/d_{\text{c}}^{\text{G}}$ was calculated to measure the effect of inhibition on RC receptive-field tuning [26, 27]. In the absence of inhibition, one would expect the relay cell to inherit the receptive-field size from the GC cell, and this ratio would be close to 1.0.

As a measure of how much the center response is reduced by the surround (center-surround antagonism), we also calculated the normalized difference between the maximum response to center stimulation (*r*_c_) and the minimum response when the surround is stimulated as well (*r*_*cs*_) [26, 27]:

$$\begin{array}{c} \alpha =\left(r_{\text{c}}-r_{\text{cs}}\right)/r_{\text{c}}\cdot 100\%. \end{array}$$ (7)

Finally, we also investigated temporal aspects of the response and computed area-response curves both for the transient (onset) response, i.e., trial-averaged spike-count firing rate for the first 100 ms after stimulus onset, and the sustained (steady-state) response corresponding to the averaged rate in the time interval from 400 to 500 ms after stimulus onset.

### Implementation

Simulation and data acquisition of the dLGN circuit model was fully implemented as class objects in Python [44], using the Python package LFPy [45] for object-representations of individual cells post-synaptic to GC units. LFPy relies on the NEURON simulation environment [33] to solve the membrane potentials for the multicompartment IN unit and single-compartment RC units. NEURON also intrinsically allows specification of neuron-to-neuron connectivity, i.e., building network models.

With a relatively low total segment count (177) for the multi-compartment IN model, each network instance was simulated serially in a matter of seconds at a temporal sampling rate of *f*_s_ = 16 kHz, resulting in realtime factors as high as ∼10% for our computer hardware described below. Parallel execution was therefore only incorporated on the parameter scan level, as discussed below. Typically, only spike times and resulting rates were returned from each network element, but readouts such as membrane voltages were readily available if needed. All simulations for each parameter set (and spot size) were repeated 10 times or more (see above) with different seeds resulting in a total of more than one million simulations.

The GC model was implemented in NEST [46] as a spike generator (rather than a neuron model) named exp_onset_generator.

Simulations were performed on a compute cluster with Intel Xeon 2 CPUs running Linux 2.6.32 using NEURON 7.3 and NEST 2.3.r10450. Software was compiled with the GNU Compiler v. 4.7.2 and linked against the GNU Science Library v. 1.14. Trials were configured using the NeuroTools.parameters package [47]. Data analysis was performed on the same computers and Apple MacBook Pro computers using NumPy 1.7.1, Pandas 0.11/0.12, and Matplotlib 1.2.1/1.3.0 under Python 2.7.3.

Results were stored in HDF5 files using the PyTables package. Further analysis was performed using Pandas/NumPy and Matplotlib for visualization.

## Results

### Synaptic integration in interneurons (INs)

**Fig 4:**
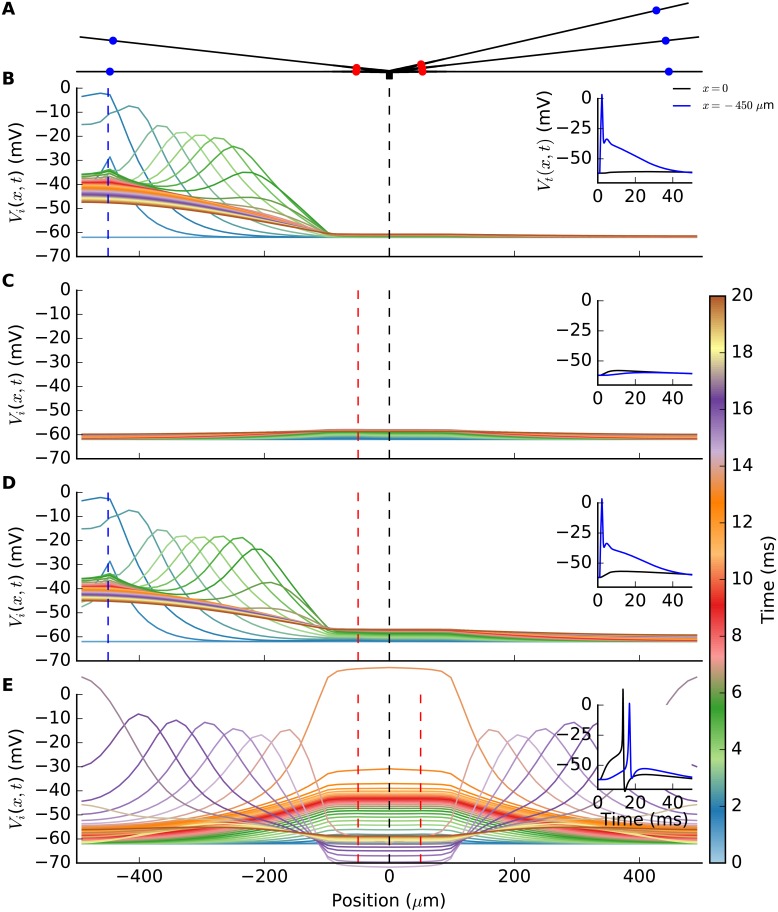
Synaptic integration properties of interneuron (IN) model. (A) Ball-and-sticks IN model consisting of a point-like soma (black square) with five dendritic sticks protruding out from it. Distal (triadic; blue dots) and proximal (red dots) synapse locations are illustrated. Panels B–E shows spatiotemporal spread of IN membrane potential along two (of five) dendritic sticks following activation by single RC spiking on inputs distal (triadic) and/or proximal synapses. Each colored line represents a snapshot of the membrane potential taken each half millisecond from 0 to 20 milliseconds with the GC spike(s) arriving at *t*_syn_ = 1 ms. The synapse position(s) are denoted by vertical, red or blue dashed lines, while the black dashed line marks the location of the soma compartment. The small inset axes show the membrane potential in the soma (*V*_*i*_(0, *t*)) and in the distal dendrite (*V*_*i*_(−450 *μ*m, *t*)), respectively, as a function of time. (B) GC spike onto distal synapse on lower left dendritic stick. (C) GC spike onto proximal synapse on lower left dendritic stick. (D) GC spike arriving simultaneously on distal and proximal synapses on lower left dendritic stick. (E) GC spikes arriving simultaneously at all five proximal synapses, including those on the two depicted dendritic sticks.

Before embarking on the dLGN circuit behavior, we demonstrate in Fig 4 the salient integrative properties of the interneuron (IN) model. The simplified ball-and-sticks morphology of the IN is illustrated in Fig 4 with the soma (black square) in the center, and the five dendrites protruding out from it with locations of both the distal, i.e., triadic, and proximal synapses marked (panel A). In the remaining panels (B–E), the membrane potential in only two selected dendrites are considered for figure clarity reasons.

When a single GC spike arrives at a distal IN synapse (panel B), the response is partly mediated by local, active ion channels. The distal dendrites undergoes a rapid, local depolarization (up to ∼0 mV) due to activation of local Na^+^ channels, after which the potential decays from subsequent activation of K^+^ (and deactivation of Na^+^) channels. The distal-dendrite membrane potential is observed to remain at a relatively depolarized level, i.e., above –50 mV, for an extended period of time (about 20 ms, see inset panel B). The endured response is partly due to the activation of local T-type Ca^2+^ channels, as we have shown previously [38]. Due to the widening of the dendritic stick, i.e., increase of stick diameter in the central direction, the EPSP is strongly attenuated upon its propagation towards the soma, and is not sufficient for driving the soma above the action potential threshold (panel B).

A single spike arriving at a proximal synapse results in only a small depolarization of the membrane potential (panel C), i.e., too little to evoke either triadic inhibition or generate a somatic action potential which in turn would provide axonal inhibition. Further, when a single distal and a single proximal synapse positioned on the same branch are activated at the same time (panel D), the resulting soma potential is still too small to generate an action potential. However, when all five proximal synapses are activated by simultaneous spikes (panel E), a somatic axon potential is generated which next provides axonal inhibition on postsynaptic RC cells. Moreover, this axonal action potential back-propagates into the dendrites where it also activates triadic inhibition. This latter type of triadic inhibition is here denoted soma-driven triadic inhibition.

### Triadic circuit dynamics

**Fig 5:**
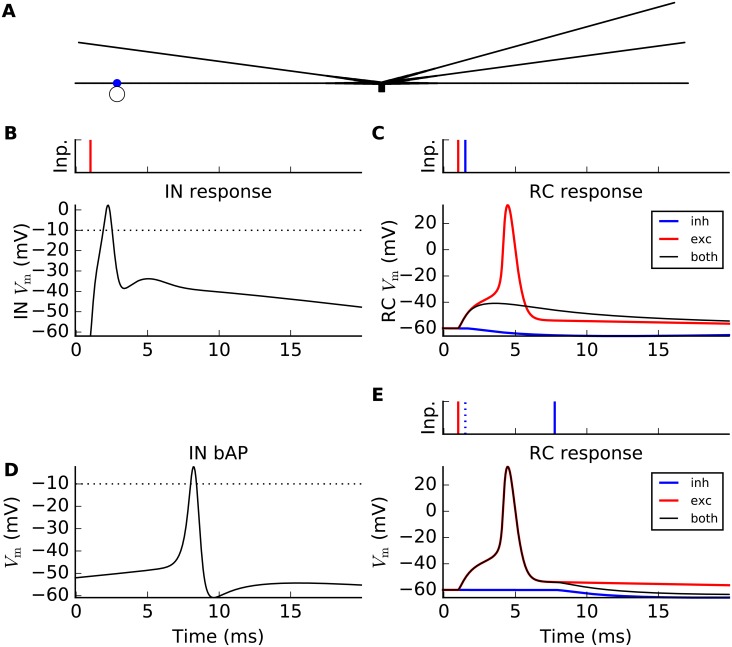
Illustration of two pathways for triadic inhibition of relay cells (RCs). Curves show membrane potentials of the IN dendrite (panels B,D) at the distal synapse position (blue dot in panel A) and in RC soma (panels C,E), respectively. (A) Illustration of interneuron (IN) with triadic connection with RC shown as open circle. (B) Single incoming GC spike input to distal (triadic) synapse (time stamp *t*_syn_ = 1 ms denoted as red bar in small display on top) triggers a large postsynaptic response in distal IN dendrite, effectively resulting in a dendritic action potential. (C) Same GC input spike as in (B) now also projecting to the RC partner of the triadic circuit with a short time delay resulting in *direct triadic inhibition* of the RC (starting at time shown as blue time-stamp bar above): without inhibition the GC input to the RC cell gives an immediate RC action potential (red curve), while no action potential occurs if the excitatory input is accompanied by direct triadic inhibition (black curve). (D) Back-propagating action potential in IN dendrite(s) triggered by a strong synapse input to the IN soma (activation time *t*_syn_ = –8 ms, *g*_max_ = 300 nS, *E*_syn_ = 10 mV, *τ* = 1 ms, *I*_syn_(*t*) = *g*_max_ ⋅ exp(−(*t* − *t*_syn_)/*τ*) ⋅ (*V*_m_ − *E*_syn_) for *t* ≥ *t*_syn_). For illustration purposes, the distal activation of the IN dendrite by the GC input is here absent, i.e., *w*_GIt_ = 0. (E) Same GC input spike as in panels B and C now also projecting to an RC cell, gives an RC action potential both without (red curve) and with *soma-driven triadic inhibition* (black curve) as the inhibition occurs too late (blue time-stamp bar above) to prevent action-potential firing in the RC.

The mechanism behind the two types of triadic inhibition, i.e., ‘direct’ and ‘soma-driven’, is illustrated in Fig 5. In panel B, a single incoming GC spike input to a distal (triadic) synapse (illustrated in panel A) triggers a large postsynaptic response in the distal IN dendrite. If the response is sufficiently large, as in the current example, it will lead to direct triadic inhibition from the IN to the RC partner in the triadic circuit. While the excitatory GC input to the RC cell alone would give an immediate RC action potential (red curve in panel C), this action-potential firing is prevented when this excitatory input is accompanied by direct triadic inhibition (black curve in panel C). (For the present model example we find that the triadic inihibition must arrive within 1.3 millisecond after the excitatory GC input to prevent the generation of an RC spike.)

In soma-driven triadic inhibition a somatic action potential in the IN, induced by sufficiently synchronous excitatory GC inputs onto the proximal dendrites (cf. Fig 4), results in a back-propagating action potential which in turn induces triadic inhibition (panel D in Fig 5). However, this type of triadic inhibition takes a few milliseconds to occur, i.e., too late to prevent the firing of an RC action potential (panel E). This inhibition can thus only affect GC spikes reaching the dLGN circuit at a later time.

Fig 5 illustrates the importance of timing of the triadic inhibition in the regulation of RC firing: when a GC spike impinges on the dLGN circuit (RC and IN cells), only the direct triadic inhibition acts fast enough to affect the immediate spike generation in RC cells. Such direct triadic inhibition probably underlies what is known as *time-locked*, or simply *locked* inhibition in the experimental literature [15].

**Fig 6:**
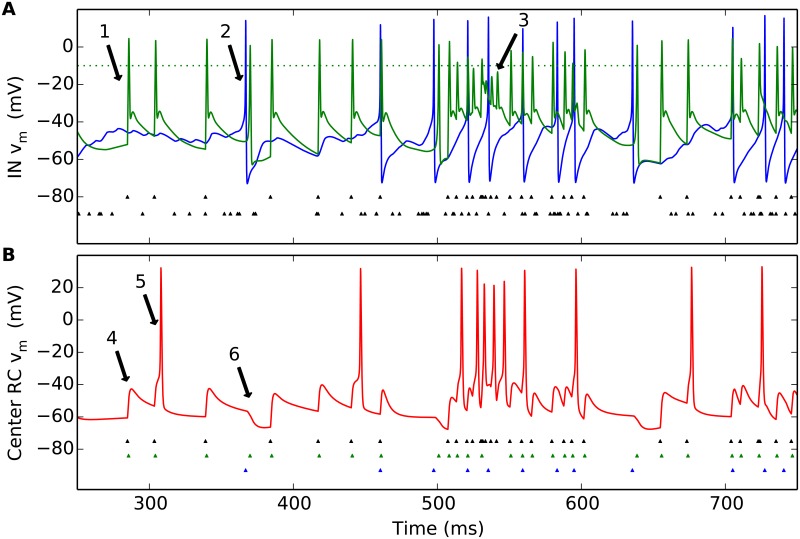
Illustration of temporal response in dLGN model circuit. A stimulus spot of diameter *d* = 1 deg is turned on at 500 ms. (A) Example of (single-trial) IN membrane-potential dynamics (soma: blue line; distal part of dendritic segment receiving synaptic input from central GC cell: green line). Also shown are GC input spikes driving the circuit, both from the center GC cell (top row of tiny triangles) and from the four peripheral GC cells (bottom row of triangles). (B) Corresponding RC membrane-potential dynamics. Also shown are input spikes from the central GC input (top row of tiny black triangles), IN dendritic (triadic) action potentials (middle row of green triangles), and IN somatic action potentials (bottom row of blue triangles). See text for explanation of arrows. Default model parameters are used, cf. Tables 2–4.

Some key features of the dynamics of the triadic circuit when stimulated by a flashing circular post, are illustrated in Fig 6. While our numerical experiments each last for 1000 milliseconds, the figure focuses on the spiking activity in the half-second window around the stimulus onset at 500 milliseconds. Panel A shows the membrane-potential dynamics of the IN for an example trial, both in the soma (blue line) and in the distal part of the dendritic segment (green) receiving synaptic input from the central GC cell. This panel also shows the time stamps of the GC input spikes driving the circuit, both from the center GC cell (top row of tiny triangles) and from the four peripheral GC cells combined (bottom row of triangles). A first observation is that in the typical case, an input spike from the central GC cell causes direct triadic inhibition (see, for example, arrow 1 in panel A) while a fairly synchronous barrage of four spikes from the set of GC cells is needed to evoke a somatic action potential (see, for example, arrow 2 in panel A). Given the much higher firing-rate of the central GC cell compared to the peripheral GC cells in the present example, the direct triadic inhibition will occur more often than firing of somatic action potentials. As a consequence, the soma-driven inhibition (soma-driven triadic and axonal) will occur less frequently than direct triadic inhibition. Note, however, that the involvement of dendritic Na^+^ and K^+^ channels in mediating the local response induces an effective refractory period (the channels do not have time to reset between two input spikes). This is evident during the first 50 milliseconds or so after stimulus onset, when the firing-rate of the central GC cell is so high that not all incoming spikes result in the distal-dendrite membrane potential passing firing threshold (see, for example, arrow 3 in panel A). Direct triadic inhibition will therefore not occur at every input spike. Such a depression of triadic inhibition for high input rates was also seen experimentally [15].

Panel B in Fig 6 illustrates the corresponding RC response. When there has been a long time since the previous excitatory GC input spike (see, for example, arrow 4 in panel B), direct triadic inhibition prevents the firing of an RC spike. However, if a new GC input spike arrives before the RC membrane potential has returned to its resting value, the direct triadic inhibition may not be sufficient to prevent the firing of an RC action potential (see, for example, arrow 5 in panel B). The chance for an incoming GC spike to generate an RC spike can be further reduced by soma-driven inhibition leading to a transiently hyperpolarized RC membrane potential (see, for example, arrow 6 in panel B). We also note that the inhibition is more efficient at preventing the firing of RC action potentials in the background state, i.e., prior to stimulus onset at 500 milliseconds, than immediately after stimulus onset: For example, during the depicted background state (250–500 ms) only two of the seven incoming GC spikes result in the firing of an RC spike, corresponding to a transfer ratio [48] of 2/7 ≈ 0.29. In contrast, in the first 75 milliseconds after stimulus onset (500–575 ms), six of thirteen incoming GC spikes result in an RC spike, corresponding to a transfer ratio of 6/13 ≈ 0.46. This transfer ratio smaller than unity value reflects that two or more incoming GC spikes are normally needed to elicit an RC spike [48–50].

**Fig 7:**
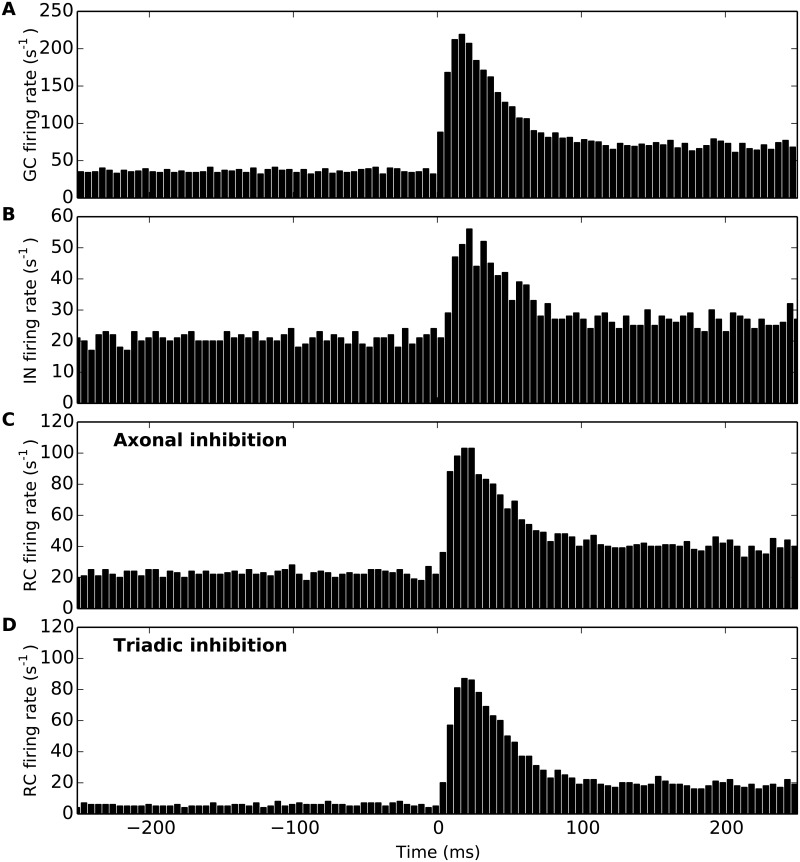
Example post-stimulus time histograms (PSTS) for cells in dLGN model circuit. Stimulus spot of diameter *d* = 1 deg is turned on at 500 ms. (A) PSTH for central GC cell. (B) PSTH for IN cell. (C) PSTH for RC cell with axonal inhibition only (*w*_IRa_ = 4 nS, *w*_IRt_ = 0). (D) PSTH for RC cell with triadic inhibition only (*w*_IRa_ = 0, *w*_IRt_ = 4 nS). Results correspond to 1000 trials, bin size: 5 ms. Default model parameters are used, cf. Tables 2–4.

As the spiking response to individual stimulus presentations typically varies between trials, the post-stimulus time histogram (PSTH) [43] is commonly used to characterize neural spiking responses. Examples of such PSTHs for the set of experiments underlying the experimental area-response curve measurements for the GC and RC on which the present model is tuned (cf. Fig 5 in [27]), can be found in [26] (Fig 3 and 4 therein). Fig 7 shows PSTHs for the GC, IN and RC cells in Fig 6 found by binning spikes found from many repetitions, i.e., many trials of our numerical ‘experiment’. Panel A shows the PSTH from the central GC cell in a 500 ms window around the spot onset, while panel B similarly shows the corresponding PSTH for the IN cells. The two lower panels show corresponding PSTHs for the RC cell for two extreme situations: only axonal inhibition (i.e., triadic inhibition turned off, *w*_IRt_ = 0) in panel C, and only triadic inhibition (i.e., axonal inhibition turned off, *w*_IRa_ = 0) in panel D. For these particular model parameters we see that the peak response in the PSTH following stimulus onset is largest for the GC (∼200 s^−1^) and smallest for the IN (∼50 s^−1^). For the RC we see that both the background (i.e., response before stimulus onset) and peak responses are larger for the case with axonal inhibition (panel C) than for triadic inhibition (panel D), implying that for the present choice of model parameters the triadic inhibition is more efficient than axonal inhibition in reducing RC firing.

### Area-summation curves

We now move on to compute and investigate area-summation curves, that is, the time-average of PSTHs of the type shown in Fig 7, as a function of spot diameter. These time-averaged PSTHs correspond to what is more precisely referred to as ‘spike-count’ firing rates [43], but in the following we will for simplicity refer to them as firing rates.

**Fig 8:**
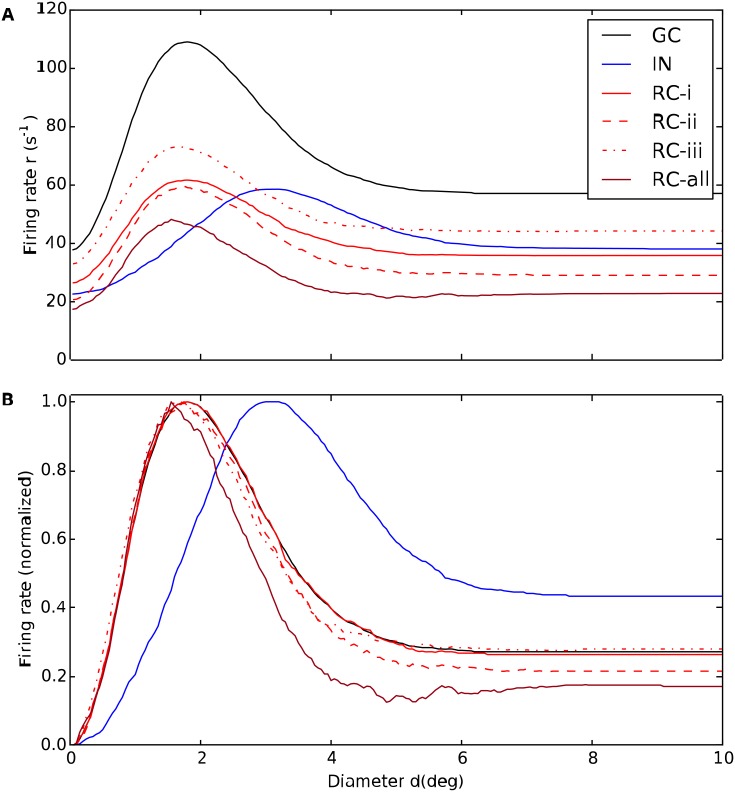
Example area-summation curves illustrating effects of various types of inhibition on relay-cell (RC) response. (A) Trial-averaged spike-count firing rate vs. spot diameter, for central retinal ganglion cell (GC, solid black), interneuron (IN, solid blue), and relay cell (RC, red lines). Red solid line: RC response for *direct triadic inhibition* (RC-i) with *w*_IRt_ = 4 nS, *w*_GIp_ = 0, *w*_IRa_ = 0. Red dashed line: RC response for *direct & soma-driven triadic inhibition* (RC-ii) with *w*_IRt_ = 4 nS, *w*_GIp_ = 0.6 nS, *w*_IRa_ = 0. Red dotted line: RC response for *axonal inhibition* (RC-iii) with *w*_IRt_ = 0, *w*_GIp_ = 0.6 nS, *w*_IRa_ = 4 nS. Dark red line (RC-all) corresponds to results from all three types of inhibition combined, i.e., *w*_IRt_ = 4 nS, *w*_GIp_ = 0.6 nS, *w*_IRa_ = 4 nS. *w*_GR_ = 15.6 nS is used in all cases. Other parameters correspond to default values. Note that the depicted IN response does not apply to case (RC-i) as the IN is only synaptically activated at the triads in this case as *w*_GIp_ = 0. (B) Area-response curves in A normalised to have maximal values of unity. The receptive-field center diameters *d*_c_ corresponds to the spot diameter giving the largest response. The spike-count firing rates are found by averaging PSTHs of the type in Fig 7 over the entire 500-ms time window the stimulus is on.

In the present modeling study we in particular investigate the effects of various types of inhibition on the area-summation curves of the RC and IN neurons. Examples of such calculated area-response curves are given in Fig 8. Here the black line gives the area-response curve of the central GC cell providing the input, the blue line the corresponding curve for somatic spikes for an IN, while the solid, dashed and dotted red lines show RC response curves for different choices of model parameters specifying inhibitory effects from the IN. The response curves shown here correspond to the ‘raw’ data, i.e., prior to filtering by a seven-point rectangular window (see Methods), and the jagged response curves serve to illustrate the inherent variability of the trial-averaged response. The bottom panel in Fig 8 shows the data normalised to the maximal response for each cell, thus highlighting the shapes of the area-response curves rather than their response magnitudes.

Fig 8 shows example area-summation curves for the three different types of inhibition considered here:

(RC-i) **Direct triadic inhibition:** Triadic inhibition driven by GC input on same distal IN dendrite only, i.e., *w*_IRt_ > 0, *w*_GIp_ = 0, *w*_IRa_ = 0
(RC-ii) **Direct & soma-driven triadic inhibition:** Triadic inhibition driven both by GC input on same distal IN dendrite and back-propagated soma activation in turn stemming from proximal inputs on the IN, i.e., *w*_IRt_ > 0, *w*_GIp_ > 0, *w*_IRa_ = 0
(RC-iii) **Axonal inhibition**: Axonal inhibition of RC following firing of action potential in the IN, i.e., *w*_IRt_ = 0, *w*_GIp_ > 0, *w*_IRa_ > 0.

The figure also shows the resulting area-summation curve when all these three types of inhibition is included at the same time.

A first observation in Fig 8 is that the GC response in all cases is larger than the RC response, essentially reflecting that the transfer ratio at the retinogeniculate relay always is less than one [26, 27, 48, 49]. The spot diameter with the largest responses corresponds to the size of *receptive-field center*, and we observe that while the central GC cell has a center diameter $d_{\text{c}}^{\text{G}}$ of about 2 degrees, the IN center diameter $d_{\text{c}}^{\text{I}}$ is about 3 degrees, cf. panel B. This larger center size reflects that the IN is driven by multiple, spatially separated GCs.

For the case with direct triadic inhibition only (RC-i) we observe that while this inhibition reduces the RC firing rate by about a factor two compared to the GC input (solid curves in Fig 8A), the shape of the response curves, i.e., normalized response, is essentially identical (panel B). Thus the direct triadic inhibition essentially acts as a gain control, only. With soma-driven inhibition included as well (RC-ii), some changes in the shape is observed (dashed red curve in panel B). In particular, a close inspection of panel B reveals that the receptive-field center size $d_{\text{c}}^{\text{R}}$ of the RC cell now is seen to be somewhat smaller than the GC center size. An even larger reduction of the center size is observed in the case of axonal inhibition only (RC-iii). This reduction in receptive-field center size seen for cases (RC-ii) and (RC-iii) (as well as the example in Fig 8 with all three types of inhibition included, RC-all) reflects the larger resulting receptive-field size of the IN providing the inhibitory action on the RC cell [26, 27].

Another key qualitative feature observed in Fig 8 is the larger *center-surround antagonism*, i.e., large relative dampening of the full-field response (e.g., *d* = 10 degrees) compared to the peak response, seen for the cases where the inhibitory effects are the strongest (RC-ii and RC-all for the example model in Fig 8). For IN this center-surround antagonism is instead reduced compared to the GC input.

In the following we show area-summation curves results both when only triadic or axonal inhibition are active like in Fig 8, and in the likely more realistic case when both types of inhibitions simultaneously affect the relay-cell response.

**Fig 9:**
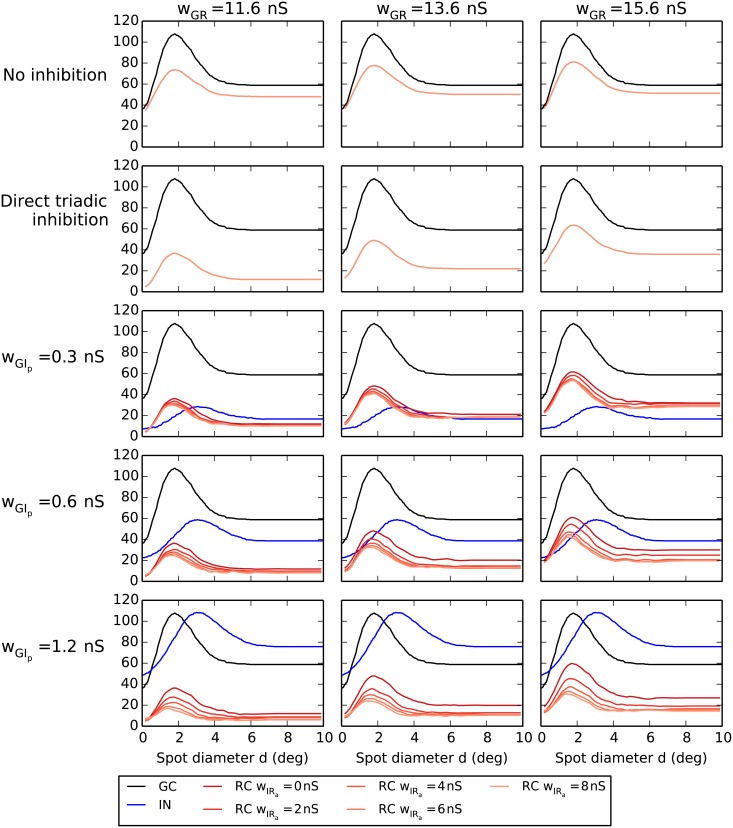
Area-response curves with triadic inhibition. Row 1: no inhibition. Row 2: direct triadic inhibition only (case (RC-i)). Rows 3–5: triadic or triadic+axonal inhibition for different values of weight proximal ganglion-cell input to the interneuron *w*_GIp_. Black curves correspond to central retinal ganglion cell (GC), blue curves to interneuron (IN), and red/orange curves to relay cell (RC). The four RC curves in the panels in rows 3–5 correspond to *w*_IRa_ = 0/2/4/8 nS with *w*_IRa_ = 0 (no axonal inhibition) and *w*_IRa_ = 8 nS (maximal axonal inhibition) corresponding to the top and bottom of the four curves, respectively.

For reference we show in the top row of Fig 9 the RC response for the case with neither triadic nor axonal IN inhibition. Here we observe that the overall RC response changes only moderately when increasing the excitatory connection strength *w*_GR_ between the central GC cell and the RC cell with almost 50% from the lowest value considered (*w*_GR_ = 11.6 nS). The reason is that the transfer ratio, i.e., the fraction of incoming GC spikes resulting in an outgoing RC spike, is already quite high even for this lowest weight. This leaves limited room for further increase in the response. Another observation is that without inhibition the RC and GC response curves always have their maxima at the same spot diameter, i.e. $d_{\text{c}}^{\text{R}}\approx d_{\text{c}}^{\text{G}}$.

With direct triadic inhibition included (second row in Fig 9) we see that the RC response curves drop substantially, e.g., about 50% for the peak response and even more for the full-field (large-spot) response for the lowest value of *w*_GR_ (11.6 nS). Unlike in the case with no inhibition, increased excitation strength *w*_GR_ is seen to increase the RC response as extra excitation will compensate for the added direct triadic inhibition. We further see that the shapes of the RC response curves are similar to the ‘no-inhibition’ curves, the main difference is a vertical shift of the response curves. Such a vertical shift implies a larger relative reduction of the full-field response compared to the center response, i.e., an increased center-surround antagonism. Thus direct triadic inhibition increases the RC center-surround antagonism *α*_R_, particularly for the lower excitatory weights. The RC receptive-field center size $d_{\text{c}}^{\text{R}}$ is essentially unaffected by the direct triadic inhibition. This follows from the fact that in our IN model, excitation of the distal IN dendrite results in small EPSP amplitudes at the soma (Fig 4B). Thus direct triadic inhibition on the RC cell can only occur due to spiking inputs from the central GC cell, and such inhibition can only affect the gain control within the triadic synapse structure (as was clearly illustrated in the normalized response plot for the direct triadic case in Fig 8B). Since distal IN excitation barely affects the somatic membrane potential and does not generate IN somatic action potentials, an IN area-summation curve is likewise absent from the second row of Fig 9.

The three lower rows of Fig 9 depict area-response curves for various combinations of direct and soma-driven triadic inhibition and axonal inhibition. The different rows correspond to different values of the proximal excitation of INs (*w*_GIp_), while different columns still correspond to different values of the retinogeniculate excitation (*w*_GR_). The area-response curve of the IN is, of course, independent of the value of *w*_GR_, so the same IN response curve is seen in the same-row panels. By comparing area-response curves for increasing values of proximal IN excitation *w*_GIp_ we see, as expected, a large increase in the IN response. The increased response is also accompanied by a reduction in center-surround antagonism of the IN neuron. However, the IN receptive-field center size $d_{\text{c}}^{\text{I}}$ is much less affected.

In each of the nine panels in the lower three rows in Fig 9 there are four (red/orange) RC area-summation curves corresponding to different values of the axonal inhibition weight *w*_IRa_. The topmost curves correspond to the situation without axonal inhibition (*w*_IRa_ = 0), while the three other curves corresponds to different non-zero values of *w*_IRa_ (2/4/8 nS) with the lowest curve corresponding to the largest weight considered (*w*_IRa_ = 8 nS). It is seen that not only does increased axonal inhibition reduce the RC response, it also reduces the RC receptive-field center size $d_{\text{c}}^{\text{R}}$. Both effects are seen to be strongest when the proximal excitation *w*_GIp_ of the IN is largest.

**Fig 10:**
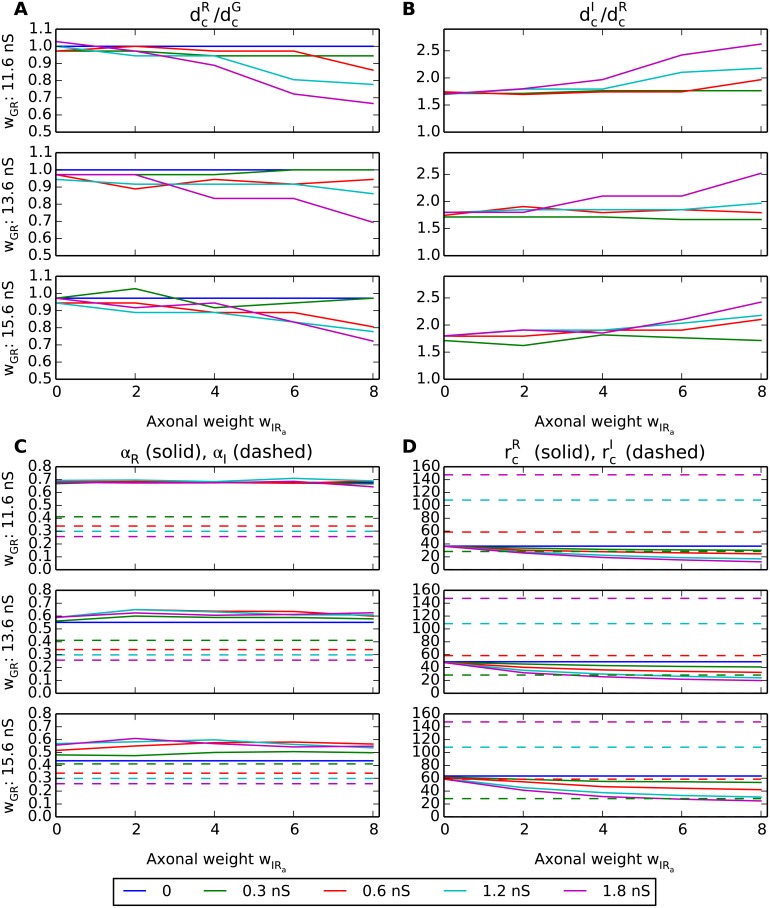
Summary of response measures from area-response curves with triadic inhibition present. (A) Ratio of receptive-field center diameter of relay cell (RC) and (central) retinal ganglion cell (GC), $d_{\text{c}}^{\text{R}}/d_{\text{c}}^{\text{G}}$; receptive-field center diameter measured as the spot diameter corresponding to the largest firing rate in the area-summation curves in Fig 9. (B) Ratio of receptive-field center diameter of interneuron (IN) and relay cell (RC), $d_{\text{c}}^{\text{I}}/d_{\text{c}}^{\text{R}}$. (C) Relay-cell *α*_R_ (solid) and interneuron *α*_I_ (dashed) center-surround antagonisms, cf. Eq 7. (D) Maximal firing rate *r*_c_, i.e., firing rate for spot exactly covering receptive-field center, for relay cell ($r_{\text{c}}^{\text{R}}$, solid) and interneuron ($r_{\text{c}}^{\text{I}}$, dashed). The colored lines correspond to different values of *w*_GIp_, see legend below panels. Note also that interneuron (IN) results are absent for the case with *w*_GIp_ = 0 (blue lines) since in this case the IN only receives triadic input and does not fire any action potentials.

The effects of the various model components and parameters on key response measures for the results in Fig 9 are summarized in Fig 10. This figure shows how the RC and IN receptive-field center sizes ($d_{\text{c}}^{\text{R}}$, $d_{\text{c}}^{\text{I}}$), center-surround antagonisms (*α*_R_, *α*_I_), and maximum firing rates vary with the axonal inhibition weight (*w*_IRa_) for a set of different values of the weight of ganglion-cell activation of the RC (*w*_GR_) and of the proximal dendrites of the IN (*w*_GIp_, color coded according to legend box below figure).

A first observation is that the receptive-field center size of the RC ($d_{\text{c}}^{\text{R}}$) is substantially reduced both when the proximal excitation of the proximal IN dendrites (*w*_GIp_) and when the axonal inhibition weight from the IN to the RC (*w*_IRa_) are increased (panel A). This is as expected as both these weights determine the overall axonal inhibition of the RC providing the shrinkage of the RC receptive-field center [27]. In contrast, the receptive-field center size of the IN ($d_{\text{c}}^{\text{R}}$) can naturally only depend on the weight of the proximal synapse from the GC (*w*_GIp_). This increase is quite modest, however, and most of the observed variation in the ratio between the IN and RC center sizes ($d_{\text{c}}^{\text{I}}/d_{\text{c}}^{\text{R}}$) (panel B) comes from the variation of the RC center size.

The center-surround antagonism for the RC (*α*_R_) is seen to be almost independent of the axonal inhibition weight *w*_IRa_ (panel C). This implies that the RC center-surround antagonism is little affected by axonal inhibition.

For the smallest values of the weight of the ganglion-cell input to the RC (*w*_GR_ = 11.6 nS), *α*_R_ does not depend much on somatic IN activity (panel C): *α*_R_ is large, about 0.7, for all considered values for *w*_GIp_. However, for the two largest values of the ganglion-cell input weight to the RC (*w*_GR_ = 13.6 nS, *w*_GR_ = 15.6 nS) some variation with *w*_GIp_ is observed: For example, for *w*_GR_ = 15.6 nS, *α*_R_ is seen to vary between ∼0.4 for *w*_GIp_ = 0 to ∼0.6 for *w*_GIp_ = 1.8 nS. As *w*_GIp_ = 0 corresponds to the case with direct triadic inhibition only (and it is also seen that *α*_R_ is essentially independent of *w*_IRa_) this substantial increase in *α*_R_ must be due to soma-driven triadic inhibition. Thus while direct triadic inhibition alone is seen to be sufficient to assure a large centre-surround inhibition when the retinogeniculate excitation *w*_GR_ is weak, soma-driven triadic inhibition can provide the same when the retinogeniculate excitation is strong.

The center-surround antagonism for the IN (*α*_I_) is generally much lower than for the RC [27] and is seen to vary between ∼0.25 and ∼0.4 depending on the value of *w*_GIp_ (panel C, dashed lines).

The maximum firing rate $r_{\text{c}}^{\text{R}}$ of the RC, i.e., the firing rate at the peak of the area-summation curve, is as expected seen to decrease both with increasing axonal inhibition weight (*w*_IRa_) and increasing synaptic input onto the proximal dendrites of IN (*w*_GIp_) (solid lines in Fig 10D). For the IN, the maximum firing rate $r_{\text{c}}^{\text{I}}$ is correspondingly seen to increase when the weight of proximal synaptic input from GCs (*w*_GIp_) increases (panel D, dashed lines).

**Fig 11:**
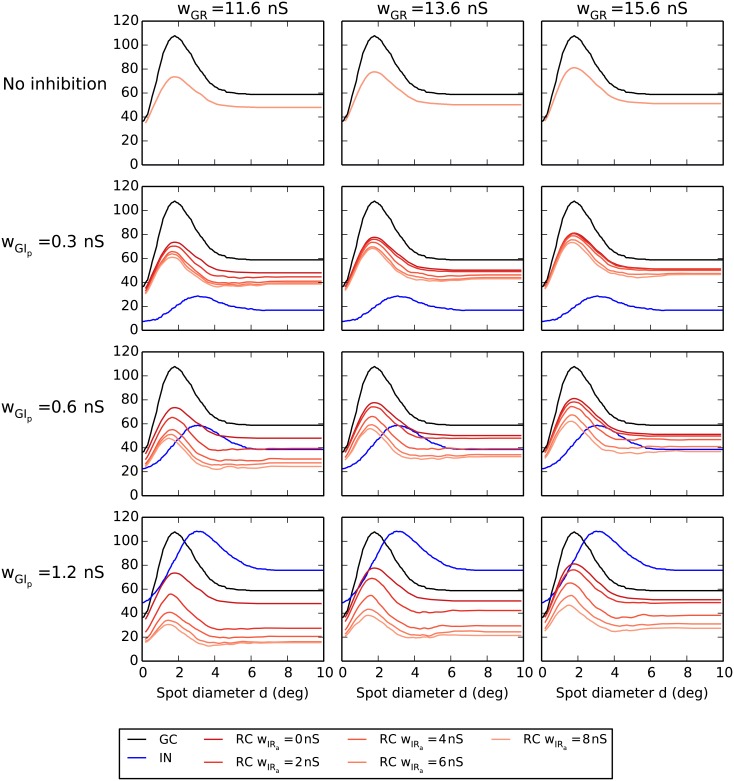
Area-response curves without triadic inhibition. Row 1: no inhibition. Rows 2–4: axonal inhibition for different synaptic weight values of proximal ganglion-cell input to the interneuron *w*_GIp_. Black curves correspond to central retinal ganglion cell (GC), blue curves to interneuron (IN), and red/orange curves to relay cell (RC). The four RC curves in the panels in rows 2–4 correspond to *w*_IRa_ = 0/2/4/8 nS with *w*_IRa_ = 0 (no axonal inhibition) and *w*_IRa_ = 8 nS (maximal axonal inhibition) corresponding to the top and bottom of the four curves, respectively.

**Fig 12:**
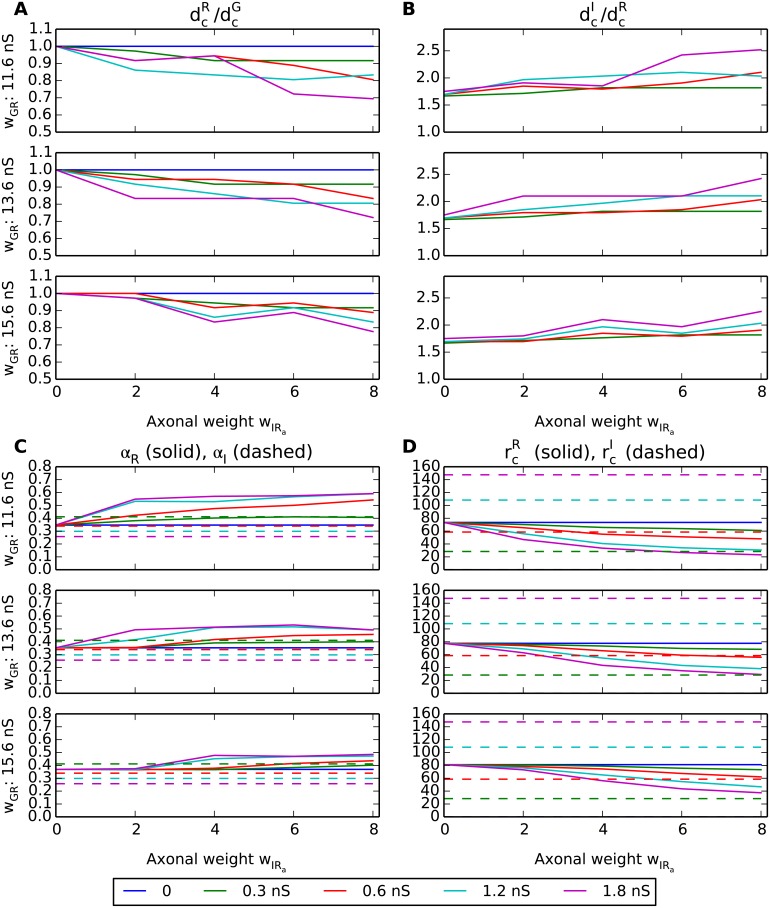
Summary of response measures from area-response curves without triadic inhibition. For explanation of panels, see caption of Fig 10.

In Fig 11 we show, in analogy to Fig 9, the same set of area-summation curves in the absence of triadic inhibition, i.e., *w*_IRt_ = 0. In this case where only axonal inhibition acts on the RCs, we observe as expected less reductions of RC responses, particularly for the smallest considered values of *w*_GIp_ and *w*_GR_. However, as confirmed by the corresponding parameter dependence of the key response measures shown in Fig 12, most qualitative effects of increasing the inhibitory synaptic weights are similar to what was seen for the case with triadic inhibition included, cf. Fig 10: The receptive-field center size of the RC ($d_{\text{c}}^{\text{R}}$) (panel A) decreases with increasing axonal inhibition (*w*_IRa_) and increasing ganglion-cell drive onto proximal IN dendrites (*w*_GIp_). This is also the case for the maximal RC firing rate $r_{\text{c}}^{R}$ (panel D), but here the firing rates are as expected overall higher compared to the case with triadic inhibition.

A final observation in Fig 12 is that the center-surround antagonism for the RC (*α*_R_, panel C) is seen to generally be lower when triadic inhibition is absent, cf. Fig 10C. This is in accordance with the previous observation for the results *with* triadic inhibition (Fig 10C) where *α*_R_ was seen to be largely independent of the axonal inhibition weight *w*_IRa_. This was interpreted to reflect dominance of triadic inhibition over axonal inhibition in determining the RC center-surround antagonism. *Without* triadic inhibition we observe in Fig 12C that *α*_R_ instead increases with the value for the axonal inhibition weight *w*_IRa_.

### Transient vs. sustained response

So far we have only considered the spike-count rate pooling all spikes within the whole 500 ms time interval following stimulus onset in our simulations. As seen in Fig 7 there is a strong *transient* component of the response with a peak in the PSTHs around 25 ms after stimulus onset while the generally much lower *sustained* (steady-state) response is reached around 100 ms after onset. This is in qualitative accordance with observations in flashing-spot experiments on cat RCs [26, 35, 51]. We thus next asked the question of whether triadic and axonal inhibition have differential effects on the transient and sustained responses of the RC.

**Fig 13:**
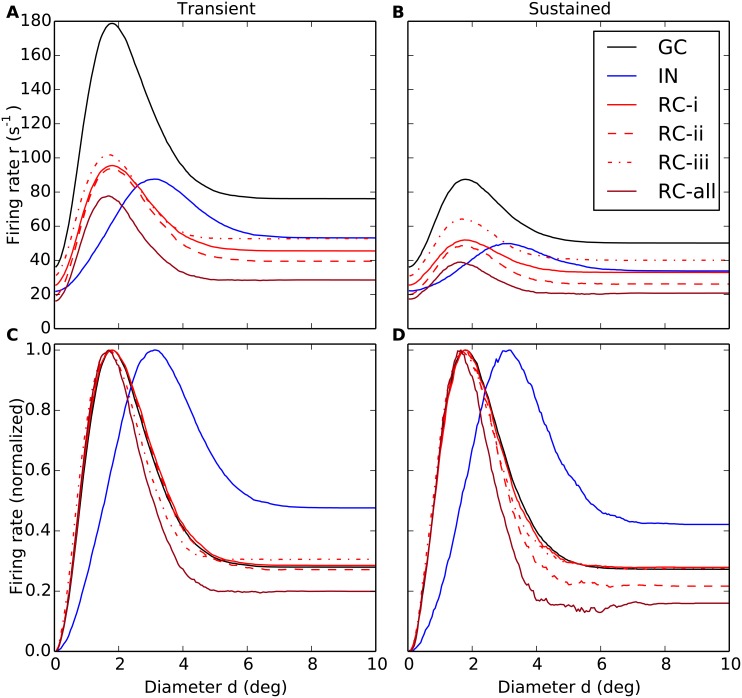
Area-summation curves for transient response and sustained response for relay-cells (RCs). (A–B) Trial-averaged spike-count firing rate vs. spot diameter, for central retinal ganglion cell (GC, solid black), interneuron (IN, solid blue), and relay cell (RC, red lines) for transient (A) and sustained responses (B). (C–D) Area-summation curves in A and B normalized to the maximum firing rate for each cell. The transient response corresponds to the trial-averaged spike-count firing rate for the first 100 ms after stimulus onset, while the sustained response corresponds to the averaged rate in the time interval from 400 to 500 ms after stimulus onset, cf. Fig 7. The depicted models examples are the same as in Fig 8: Red solid line: RC response for *direct triadic inhibition* (case (RC-i)) with *w*_IRt_ = 4 nS, *w*_GIp_ = 0, *w*_IRa_ = 0. Red dashed line: RC response for *direct & soma-driven triadic inhibition* (case (RC-ii)) with *w*_IRt_ = 4 nS, *w*_GIp_ = 0.6 nS, *w*_IRa_ = 0. Red dotted line: RC response for *axonal inhibition* (case (RC-iii)) with *w*_IRt_ = 0, *w*_GIp_ = 0.6 nS, *w*_IRa_ = 4 nS. Dark red line (RC-all) corresponds to results from all three types of inhibition combined, i.e., *w*_IRt_ = 4 nS, *w*_GIp_ = 0.6 nS, *w*_IRa_ = 4 nS. *w*_GR_ = 15.6 nS is used in all cases. Other parameters correspond to default values. Note that the depicted IN response does not apply to case (RC-i) as the IN is only synaptically activated at the triads in this case as *w*_GIp_ = 0. Note also that 500 trials, not the default value of 10 trials, were used to compute each depicted trial-averaged spike-count rate, and that no seven-point filtering was employed to smooth the area-summation curves.

**Fig 14:**
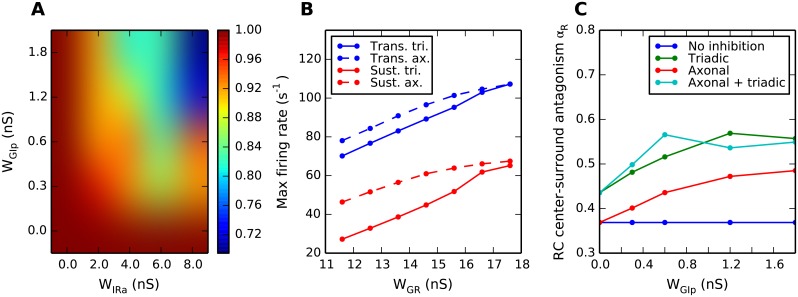
Summary of key results on different effects of triadic and axonal inhibition on relay-cell (RC) response. (A) Dependence of diameter of RC receptive-field center $d_{\text{c}}^{\text{R}}$ on two key model parameters (*w*_GIp_, weight of proximal excitation of the interneuron (IN); *w*_IRa_, weight of axonal inhibition) for the case of axonal inhibition only (i.e., *w*_IRt_ = 0). For this example the diameter of the ganglion-cell receptive-field center $d_{\text{c}}^{\text{G}}$ is fixed to 1.8 deg, and the retinogeniculate excitation is set to *w*_GR_ = 11.6 nS. (B) Transient and sustained RC responses for center-filling spots, corresponding to maximal responses in the area-response curves, for the cases of only triadic or only axonal inhibition. Dependence of maximal response on retinogeniculate excitation weight *w*_GR_ is depicted. Other parameters: *w*_IRa_ = 4 nS, *w*_GIp_ = 0.6 nS. (C) Dependence of center-surround antagonism, quantified by the coefficient *α*_RC_ (Eq 7), on *w*_GIp_, the weight of the GC excitation of INs on the proximal dendrites. Dark-blue line: No inhibition, *w*_IRa_ = *w*_IRt_ = 0. Red line: Axonal inhibition only, *w*_IRt_ = 0, *w*_IRa_ = 8 nS. Green line: Triadic inhibition only, *w*_IRa_ = 0. Light-blue line: Both triadic and axonal inhibition, *w*_IRa_ = 8 nS. Retinogeniculate excitation is set to *w*_GR_ = 15.6 nS. In B and C simulation data points are marked with dots, and lines are added as a guide for the eye. Note that 500 trials, not the default value of 10 trials, were used to compute the depicted trial-averaged spike-count rate in panel B.

In Fig 13 we compare area-response curves computed for the transient phase (0–100 ms after stimulus onset) to the sustained phase (400–500 ms after stimulus onset) for the same model examples as in Fig 8. Comparison of the (unnormalized) responses in the top row demonstrates the large differences in firing rates, the transient response (panel A) being up to a factor two larger than the sustained response (panel B). For the present model examples, the triadic and axonal inhibition are seen to be roughly equally effective in dampening the RC response for the transient response for spots filling the receptive-field center (panel A). Interestingly, however, the triadic inhibition is seen to be more effective than axonal inhibition in dampening this response to center-filling spots for the sustained response. This feature is seen also for other values of retinogenculate excitation *w*_GR_ than the one used in this example, cf. Fig 14B.

Comparison of the normalized area-response curves (panels C and D in Fig 13) reveals only subtle differences in the area-response shapes. One observation is that soma-driven triadic inihibition seems slightly more effective in suppressing the sustained than the transient RC responses for the largest spot diameters.

For the sustained response we also observe in panel D a weak ‘noisy’ minimum in the response for spot diameters *d* around 5–6 degrees for the case with both triadic and axonal inhibition (RC-all), a feature not present for the transient response (panel C). This minimum stems from the strong activation of the INs for these spot sizes (cf. blue curve in panel D) compared to for the larger spot sizes, i.e., *d*∼8–10 degrees. While this feature of the IN response curve is also present for the transient response, it is slightly less so. The more prominent role of the combined triadic and axonal inhibition in modifying the receptive-field of the RC (compared to the GC) in the sustained response than in the transient response is also manifested by the slightly smaller receptive-field center size ($d_{\text{c}}^{\text{R}}$) and widths of the peak of the area-summation curves, cf. panels C and D in Fig 13.

## Discussion

In the present modeling work we have investigated putative roles of triadic and axonal inhibition from dLGN interneurons (INs) on the visual response properties of dLGN relay cells (RCs) relaying visual information to cortex. Taking advantage of a recently developed biophysically detailed multicompartmental model for an IN, the study is the first investigating the effects of different inhibitory actions of INs, i.e., triadic inhibition due to dendrodendritic contacts between INs and RCs and standard axonal inhibition. The interplay of these two inhibitory pathways is expectedly essential for understanding the functional role of inhibition in the dLGN circuit [52, 53]. To compare with (i) *in vivo* data of responses to flashing spot stimuli for cat RC cells of the X-type [26] and (ii) previous firing-rate models of the dLGN circuits [27], we have focused on a minimal network motif consisting of five RCs, modeled as single-compartment neurons, receiving inhibition from a single multicompartmental IN. These dLGN neurons are in turn driven by spiking inputs from five neighbouring retinal ganglion cells (GCs).

As in [27] we have computed and investigated area-response curves for circular flashing spots and studied how the model ingredients and parameters affect their detailed shapes and features, in particular (i) the *receptive-field center size d_c_* of RCs and INs, identified as the flashing spot diameter giving the largest spike-count firing-rate reponse, (ii) the *maximal firing rate* (occuring for spots exactly filling the receptive-field centers), and (iii) the *center-surround antagonism α* measuring the suppression of large-field responses compared to the maximal responses. A particular focus of our study has been the investigation of differential effects of triadic and axonal inhibition on the response properties of RCs, and key findings are summarized in Fig 14 and discussed below.

### Triadic vs. axonal inhibition

Our model contains three distinct types of inhibition, (i) direct triadic inhibition, (ii) soma-driven triadic inhibition and (iii) axonal inhibition, each with putatively different inhibitory effects on the RCs.

#### Gain control vs. shaping of RC spatial receptive fields

One of the findings is the different roles played by direct triadic and axonal inhibition in shaping the spatial receptive fields of RCs. Direct triadic inhibition was found to essentially only provide a simple non-linear gain control of the conversion of input spikes to output spikes by RCs (cf. two top rows of Fig 9), with no qualitative changes on the shape of the area-response curve (cf. RC-i in Fig 8B). The receptive-field center size was unchanged compared to the no-inhibition situation, reflecting the assumed spatially local inhibitory action on a single dendritic branch.

Axonal inhibition, on the other hand, was found to have a substantial effect on the size of the receptive-field center: the larger the inhibition, the more the RC center size shrinks compared to the center size of the retinal ganglion cell (GC) providing the feedforward excitation, see, e.g., panel A of Fig 12. This shrinking follows from the larger receptive-field center size of the IN compared to the central GC [26, 27]. Thus the exact magnitude of the receptive-field shrinking will in our model depend on the parameter values for the proximal GC excitation of INs (*w*_GIp_) and the weight of the axonal inhibition of the central RCs by the IN (*w*_IRa_) as shown in Fig 14A. These results for the ratio $d_{\text{c}}^{R}/d_{\text{c}}^{G}$ show that the product of these two model parameters is a good predictor of the receptive-field shrinkage resulting from axonal inhibition.

As soma-driven triadic inhibition is activated by the same IN action potentials that drive axonal inhibition, this type of triadic inhibition will also contribute to the shrinking of the RC receptive-field center. This is for example observed for the example with both direct and soma-driven triadic in Fig 8B (RC-ii) where the receptive-field center is smaller than for direct triadic inhibition only (RC-i) (but larger than for axonal inhibition only, RC-iii).

Thus, a possible role of the different inhibitory actions from INs to RCs in the dLGN circuit is to provide separate mechanisms for affecting gain control only (direct triadic inhibition) and regulation of receptive-field center size (axonal inhibition, soma-driven inhibition) of visual signals sent to cortex. A smaller receptive-field center size suggests higher spatial resolution, but see [54].

#### Center-surround antagonism

The center-surround inhibition has been observed to be larger for LGN RCs compared to the retinal GCs driving them, see [26] and references therein. Both triadic and axonal inhibition contribute to increasing the RC center-surround antagonism compared to GC input, but triadic inhibition was found to be the most efficient mechanism in doing so. This is further illustrated in Fig 14C where we compare the center-surround antagonism coefficient *α*_R_ measuring the dampening effect of the inhibitory surround on the RC response compared to the maximal RC response (Eq 7). With axonal inhibition only, *α*_R_ increases from the no-inhibition value of 0.37 to 0.49 found for the maximal values considered for *w*_GIp_ (1.8 nS) and *w*_IRa_ (8 nS) in this study. With direct triadic inhibition only (i.e., triadic-inhibition curve for *w*_GIp_ = 0), *α*_R_ is at a modest value of 0.44. With soma-driven triadic inhibition included as well (i.e., triadic-inhibition curve for *w*_GIp_>0), *α*_R_ gradually increases with increasing excitation of the IN (i.e., increasing value for *w*_GIp_) up to a maximum value of *α*_R_ = 0.56. Interestingly, addition of axonal inhibition on top of this triadic inhibition is seen in Fig 14C to have little effect on the total value of *α*_R_, even with our maximum weight for the inhibition of the RC (*w*_IRa_ = 8 nS). Thus while direct triadic inhibition alone is seen to be sufficient to assure some extra centre-surround inhibition, the addition of soma-driven triadic inhibition increases *α*_R_, substantially. Note, however, that the model example in Fig 14C has a fairly strong retinogeniculate excitation, i.e., *w*_GR_ = 15.6 nS. For weaker retinogeniculate excitation, e.g., *w*_GR_ = 11.6 nS, we observed in Fig 10C that direct triadic inhibition alone was sufficient to provide a large value of center-surround antagonism.

#### Transient vs. sustained response

Another difference between the effects of axonal and triadic inhibitions were observed when comparing area-response curves computed for the transient phase (0–100 ms after stimulus onset) with corresponding responses in the sustained phase (400–500 ms after stimulus onset) (Fig 8). As found for the example in Fig 13, triadic inhibition seems, compared to axonal inhibition, to be particularly efficient in inhibiting the sustained RC response. As demonstrated in Fig 14B this is also observed for other values of retinogeniculate excitation, parameterized in our model by *w*_GR_, and is particularly prominent for weaker excitation. Here the maximum RC response, i.e., occurring for spots filling the receptive-field center, is shown both for the transient and sustained responses for different values of the retinogeniculate excitation *w*_GR_. Only the cases with direct triadic inhibition or axonal inhibition (cases (RC-i) and (RC-iii)) are considered. For all values of *w*_GR_ less than 15.6 nS, the value used in the example in Fig 13, we see that the reduction of the sustained response compared to the transient response is particularly prominent for the case with triadic inhibition only.

#### Timing of inhibitory action

Direct triadic inhibition where time-locked dendrodendritic inhibition occurs due to the same GC spike that provides the excitatory input, was the fastest-acting of the three types of inhibition presently considered. Both soma-driven triadic and axonal inhibition follow only after the generation of IN action potentials which, in our model, required multiple simultaneously arriving GC spikes at the proximal dendrites of the IN. Following this simultaneous input volley it took in our model on the order of ten milliseconds for the action potential occur, a time lag mainly set by the time needed to activate the calcium currents involved in driving the neuron above the action-potential initiation threshold. Thus effects both from soma-driven triadic and axonal inhibition are substantially delayed compared to putative excitatory effects of the same GC input spikes. In our model the time constant of activation of the axonal inhibition was on the order of 1 ms (cf. Table 3), much shorter than the time it takes for the IN action potential to backpropagate from the IN soma to the triad and activate soma-driven triadic inhibition (∼5 ms, cf. Fig 5). Thus axonal inhibition was a faster mode of inhibition than soma-driven triadic inhibition in our model.

#### Role of soma-driven inhibition

Finally, it should be noted that of the three types of inhibition, soma-driven triadic inhibition is both conceptually and biophysically less understood than the two others: Although somatically elicited APs in INs are known to successfully invade distal dendrites [37], it has still not been proven that they actually do trigger dendritic GABA release directly, although this seems likely [41]. It might nevertheless be that the key role of soma-controlled dendritic signals is to regulate the release probability, e.g., via the activation of local dendritic NMDA receptors [41] or metabotropic glutamate receptors [42]. These mechanisms were not included in the current model, and more experimental studies are clearly needed to constrain this aspect of the model.

### Future model applications and extensions

We consider the present investigation to be only the first of several applications of the present modeling approach. Until recently, dLGN circuit models lacked a key ingredient, namely an IN model incorporating the key dual-action inhibitory features of this cell type. With the arrival of the first multicompartmental dual-action IN model [24], we can now, with the combined use of existing (single-compartment) Hodgkin-Huxley type models for relay cells (see [55] for an overview), investigate dLGN circuitry in models at a new level of biological realism. This will not only enable elucidation of the role of dLGN circuitry in shaping spatial response features like here, but also the key role played by the circuit in temporal processing of the incoming spike trains from retina [39, 52]. Below we discuss various directions where the present modeling approach should be considered.

#### Brain state—tonic vs. bursty spiking

The neurons in the dLGN receive modulatory input from other parts of the brain which, among other things, may cause the dLGN circuit to shift between drowsy and attentive cognitive states [56, 57]. Such state-regulating modulation can shift the resting membrane potential of both RCs and INs, which in turn can switch the firing mode of these neurons between tonic and bursty [13, 39, 56–60]. In the current study, our RC and IN models were based on data from RC and IN neurons that both rested on relatively depolarized membrane potentials (–60 mV and –63 mV, respectively) and were characterized by tonic response modes, cf. Fig 2. One obvious step in future investigations would be to explore how the conclusions we arrived at in the current work depend on the processing state of the dLGN.

#### Firing regime and receptor activation

The signal processing in the dLGN depends in non-trivial manners on external conditions. In [14], the author distinguishes between two input regimes in the dLGN: (i) a low-input regime (low GC firing frequency), where the glutamatergic input to relay cells and INs is thought to be primarily mediated by AMPA receptors, and (ii) a high-input regime that also triggers the slower NMDA and metabotropic glutamate receptors (mGluRs). Although recent experiments have indicated that a sharp distinction between low and high-input regimes may be questionable [61], it is still likely that the input intensity determines which receptor subtype that dominates in generating the dLGN response. Our model was adapted to experimental data where the observed IN and RC responses were found not to depend on mGluR-activation [15, 41]. The model predictions are therefore expectedly most valid for signal processing in the low-input regime. However, mGluR activation could have numerous additional effects on the signal processing in the dLGN [12, 42, 61–63], and a natural step in future investigations would be to investigate effects of mGluR activation in the current model.

A subclass of RC cells, the so-called *lagged* cells, has been found to have delayed visual-response onset and an initial suppression of response until it reaches a maintained firing level [5], contrasting the fast and strong transient response found for the presently studied non-lagged cells, cf. the PSTHs in panels C and D in Fig 7. This property has been linked to dominance of NMDA receptors over AMPA receptors at the excitatory part of the retinogeniculate synapse [64]. Again, the effect of various combinations of these ionotropic glutamate receptors at this synapse could directly be studied in a modified version of our model.

#### Retinal input

In the present model application we have focused on the situation with a single dominant retinal GC input to each RC as in the analogous firing-rate based model in [27]. RCs receiving input from multiple GCs have also been observed [7, 8]. However, our present model assumption of a single GC input naturally accounts for the experimental observation that the receptive-field centers for the RCs typically have similar or smaller sizes than those of the GCs (as measured by S-potentials) [26]. With strong inputs from several spatially displaced GCs, the receptive-field centers of the RCs would instead be larger than those of the GCs and thus at odds with the experimental data set on which the present version of the model is tuned. The study of the effect of having multiple GC inputs would be a natural future application [65], as would the study of the effect of having a set of discrete units describing the receptive field of the GC rather than the circularly symmetric DOG model [66].

Further, the present retinal input was described by a spatiotemporally separable filter model, i.e., the spatiotemporal GC receptive field was assumed to be described by a spatial function (in this case a DOG-model [36]) multiplied by a temporal function. However, several studies have demonstrated receptive fields in the GCs driving the LGN which are non-separable in space and time [51, 67, 68]. In [35] a new non-separable spatiotemporal receptive-field model, consisting of a sum of a transient and sustained component, was derived based on high-resolution, time-resolved area-summation curves for LGN RCs from [51]. As qualitatively similar area-summation curves was observed for the GC input, a future project would be to explore the LGN circuit response to GC input described by such non-separable filter models.

In our model application we have also assumed a (time-modulated) Poissonian distribution of the incoming GC spikes. Experimental recordings have revealed a more regular spike-train input than Poissionian [48, 69, 70]. The effect of this regularity could be investigated by considering input GC spike trains instead obeying a gamma-process statistics, as in the model study of [50].

#### Feedback from cortex and thalamic reticular nucleus (TRN)

Both RCs and INs receive excitatory feedback from cortex, both via ionotropic and metabotropic receptors, and inhibitory feedback from the GABAergic neurons in the thalamic reticular nucleus [11, 39, 71–74]. The effect of the feedback on circuit behaviour will likely depend strongly on whether the feedback arrives on distal dendrites or close to the soma. This is again a question that can be directly investigated with a network model of the present type including a spatially extended multicompartment IN model incorporating both triadic and axonal actions.

Local dendritic processing is particularly important in the electrotonically extensive INs [28], but recent experiments have indicated it also plays an important role in processing cortical input to distal RC dendrites [75]. In the current study, we explored the LGN response to retinal input, and kept a particular focus on the processing by the triadic synapse which tends to be located distally at IN dendrites and proximally at RC dendrites. We therefore used an extensive, multicompartmental IN model, while we assumed that a single-compartment model was sufficient for the (generally more compact [28]) RC. However, to properly explore modulatory input to LGN arriving at distal dendritic sites, it is likely that our model should be expanded to include a more comprehensive, multicompartmental model also for the RCs (see, e.g., [23]).

#### Synaptic plasticity

The present model assumes static synapses while *in vitro* studies have demonstrated short-term synaptic plasticity throughout the circuit. The feedforward excitatory synapse from GCs to RCs has been found to be short-term depressing [76] as has the feedback excitatory synapse from cortical cells to INs [73]. In contrast, the feedback excitatory synapse from cortical cells to RCs is short-term facilitating [77]. This suggests the possibility for interesting and varied dynamics in the dLGN circuit, a dynamics that likely will require detailed circuit modeling to unravel.

#### ‘Same-sign’ vs. ‘push-pull’ inhibition

In the present model the IN has been assumed to receive GC input with symmetry of the ‘same sign’, i.e., ON or OFF, as the RC it inhibits. For the direct triadic input this ‘same-sign’ follows directly, but it is also possible that the proximal synaptic input to the IN which drive the generation of IN action potentials, has the opposite ‘push-pull’ symmetry [6, 78]. If so, the axonal inhibition (as well as the soma-driven triadic inhibition) from the IN onto the RC will have the opposite symmetry compared to the excitatory input to the RC from the GC, an arrangement suggested in [53, 65, 79]. While this arrangement seems difficult to reconcile with the observed shrinking of the RC receptive-field center compared to the GC input [26, 27], the consequences of driving IN action-potential firing with proximal GC inputs with opposite or mixed symmetries (cf. [65]), should also be explored in a model of our type.

### Outlook

The present model is based on data from several animal species: the target RC and GC area-response curves are from cat dLGN [26, 27], the RC single-compartment neuron model was developed to investigate network dynamics in ferret slices [29], while the multicompartment IN model is based on data from mice [24]. Main features of thalamic physiology seem to be well conserved across species [52]. However, the applicability of the present ‘chimeric’ model to account for data from different species is presently unknown, in particular since different arrangements of the LGN circuit elements may give very different signal-transformation properties [65, 78]. This will have to be explored by comparison of model predictions with experimental data from the various species.

With the advent of ever more sophisticated techniques for controlling gene expression in mice (accompanied by the possibility for optogenetic activation [80]), the mouse dLGN has emerged as a particularly interesting model system [52]. The full mouse dLGN has only about 18.000 neurons, so network simulations of a sizable fraction of the visual field is feasible with present-day computers. However, ‘no nucleus is an island’ [52], and a comprehensive understanding of the function of the dLGN circuit likely also will require simultaneous modeling of the primary visual cortex (with 360.000 neurons [81]) and maybe also other brain areas. Such modeling of the visual thalamocortical system in mice can be facilitated by joint application of models at different levels of resolution. In the present model, for example, the GC input was modeled by means of stochastically generated spike trains obeying spatiotemporal probability distributions found from descriptive firing-rate models. The RC cells were modeled as single-compartment Hodgkin-Huxley type neuron models producing spikes, but the connection to the previous firing-rate model of the same system [27] was apparent as very similar trial-averaged area-response curves were produced. In the same vein one could envision modeling the effects of cortical feedback to the dLGN circuit by means of firing-rate models for populations of cortical cells feeding back to spiking network models in the dLGN (rather than firing-rate models [74]). With a comprehensive mapping of the physiological and anatomical properties of the cells (and their connections) in mouse dLGN and visual cortex on the way [81, 82], the time seems ripe for comprehensive efforts to finally build mechanistic models mimicking signal processing in the dLGN.
